# EGCG and DOX dual-drug-loaded enzyme-responsive nanovesicles boost mitochondrial-mediated ICD for improved immunotherapy

**DOI:** 10.3389/fphar.2025.1624109

**Published:** 2025-07-07

**Authors:** Mengxue Zhou, Ying Wang, Hongchun Cui, Huan Geng, Lifo Ruan, Yanni Zhao, Chuang Zhou, Weidong Dai, Jun Chen, Jizhong Yu, Haipeng Lv, Zhi Lin

**Affiliations:** ^1^ Key Laboratory of Tea Biology and Resource Utilization of Ministry of Agriculture, Tea Research Institute, Chinese Academy of Agricultural Sciences, Hangzhou, China; ^2^ Tea Research Institute, Hangzhou Academy of Agricultural Science, Hangzhou, China; ^3^ Department of Orthopedic Surgery, The Second Affiliated Hospital, Zhejiang University School of Medicine, Hangzhou, Zhejiang, China; ^4^ CAS Key Laboratory for Biomedical Effects of Nanomaterials and Nanosafety, Multi-disciplinary Research Division, Institute of High Energy Physics and University of Chinese Academy of Sciences, Chinese Academy of Sciences, Beijing, China; ^5^ School of Food and Biological Engineering, Shaanxi University of Science & Technology, Xi’an, China

**Keywords:** MMP-2 sensitive nanovesicles, EGCG, tumor fibrosis, mitochondria-targeted, cancer immunotherapy

## Abstract

Enhancing cancer immunotherapy using methods that induce immunogenic cell death (ICD) can significantly improve its effectiveness and profoundly influence its role as a highly efficient cancer treatment strategy. However, the limited penetration of cytotoxic T cells into tumors, owing to dense tumor fibrosis, remains a significant barrier to immunotherapy. A tumor microenvironment-sensitive intelligent dual-drug delivery system was developed to simultaneously deliver epigallocatechin-3-gallate (EGCG) and doxorubicin (DOX) to mitochondria. EGCG enhanced the mitochondria-targeted action of DOX and increased damage to the mitochondrial electron transport chain which facilitated capturing electrons in the mitochondrial matrix of DOX. Subsequently, DOX molecules form a semiquinone intermediate and electrons are transferred to oxygen to generate reactive oxygen species (ROS) that induce mitochondrial apoptosis. These results indicate that EGCG amplifies the combined effects of chemo/chemodynamic therapy of DOX, demonstrating a pronounced synergistic ICD effect that recruits CD8^+^ T cells to the tumor microenvironment (TME). In addition, EGCG promotes T-cell infiltration into tumor tissues by inhibiting the transforming growth factor-β signaling pathway, thereby significantly enhancing antitumor efficacy. This study advances the efficacy of immunotherapy through bidirectional synergy, which not only enhances intrinsic tumor immunogenicity but also overcomes the extrinsic physical barriers of tumors, providing a new direction for the development of broadly applicable immunotherapies.

## 1 Introduction

Immunogenic cell death (ICD) establishes a groundbreaking framework for contemporary tumor immunotherapy. This form of cellular death results in the release of factors that activate the immune system (Liu et al., 2019; Wang et al., 2020; Fu et al., 2022). Mechanistically, ICD is initiated by the release of damage-associated molecular patterns, such as calreticulin (CRT) and high-mobility group box 1, from dying tumor cells (Hou et al., 2024; Shi et al., 2024). This process promotes phagocytosis of tumor antigens by antigen-presenting cells (APCs), thereby efficiently initiating a targeted antitumor immune response (Chen et al., 2023; Zhao et al., 2024; Dou et al., 2025). Numerous chemotherapeutic agents (such as doxorubicin [DOX] and oxaliplatin), radiation therapy, and photodynamic therapy have been shown to induce ICD by enhancing endoplasmic reticulum (ER) stress (Zheng et al., 2022; Pan et al., 2023). The production of intracellular reactive oxygen species (ROS) may as a critical requirement and fundamental component of ICD induction. Consequently, researchers have hypothesized that oxidative stress in specific organelles within tumor cells may play a pivotal role in initiating ICD (Guo et al., 2022; Zhu et al., 2022). Mitochondria are crucial organelles that not only serve as energy reservoirs for cellular functions but also fulfill numerous regulatory roles in apoptosis, making them a promising target for immunotherapy (Li et al., 2022; Luo et al., 2022). In recent years, extensive studies have focused on enhancing mitochondria-mediated ICD, which has led to the development of various innovative therapeutic approaches. Chen et al. (2019) investigated the relationship between mitochondrial oxidative stress and ICD, clarifying the mechanism by which mitochondrial ROS-mediated oxidative stress triggers ICD in tumor cells, and revealed a strong correlation between the two (Chen et al., 2019). This evidence provides a framework for initiating ICD through the modulation of mitochondrial function, whereas integrated cancer immunotherapy presents broadly applicable strategies to elicit enhanced antitumor immune responses (Peng et al., 2023; Liu et al., 2024; Qian et al., 2024).

Because T cells must directly engage with tumor cells to achieve effective antitumor activity, tumor cells can evade these attacks using physical shields (Pinter and Jain, 2017; Pan et al., 2022; An et al., 2023). These physical or biological barriers contribute to the formation of immunosuppressive tumor microenvironments (ITM) that exclude immune cells (Marigo et al., 2016; Ni et al., 2020; Lu et al., 2023). Although various strategies have been developed to enhance tumor immunogenicity, a notable deficiency is the lack of infiltration by CD8^+^ T cells or dendritic cells (DCs), which undermines the efficacy of immunotherapy (Hanoteau et al., 2019; Trujillo et al., 2019). Furthermore, some ICD inducers may not elicit effective antitumor immunity when conventional administration strategies (Gao et al., 2008; Nam et al., 2018; He et al., 2021). For instance, therapeutic agents may lose their medicinal efficacy under physiological conditions, exhibit insufficient circulation time, demonstrate poor accumulation within tumor tissues, have inadequate penetration into intratumoral regions, and rely on passive diffusion, leading to potential harm and toxicity to normal cells. Consequently, the development of mitochondria-targeted delivery systems and innovative nanostructured molecules that can overcome the delivery barriers of ICD inducers is a promising strategy to enhance immunotherapy.

**FIGURE 1:**
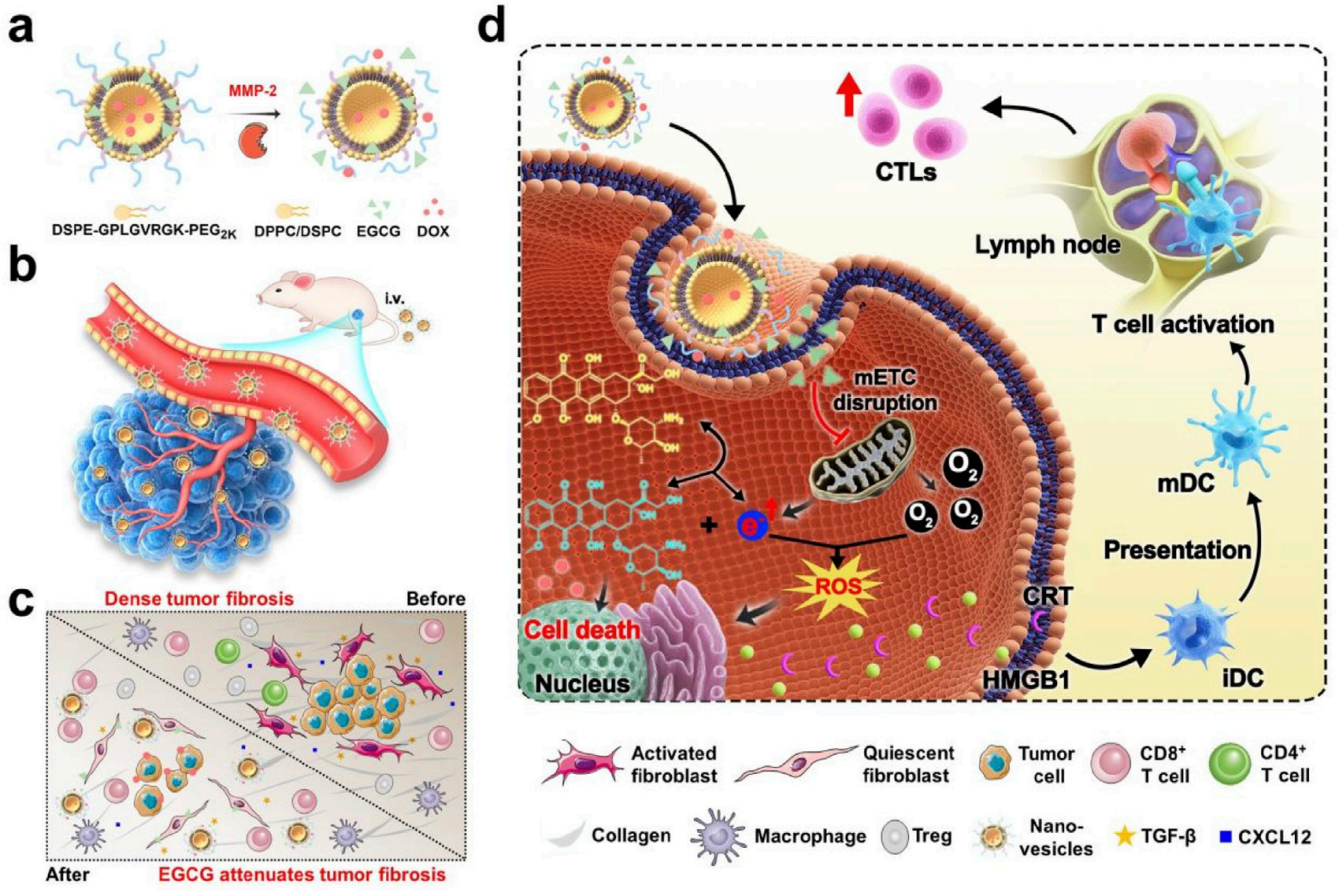
EGCG- and DOX-loaded tumor microenvironment-responsive nanovesicles augment the efficacy of immunotherapy against TNBC by enhancing intrinsic tumor immunogenicity and overcoming the extrinsic physical barrier of the tumor. **(a)** Schematic drawing of the dual-drug-loaded nanovesicles and enzyme-triggered drug release process. **(b)** Nanovesicles-mediated deep penetration of DOX. **(c)** Nanovesicles-mediated improvement in the immune cells infiltrating. **(d)** Schematic of the working mechanism of the EGCG synergizes with DOX to enhance the tumor immunotherapy.

We constructed enzyme-triggered dePEGylation nanovesicles (designated EEDNV) composed of 1,2-dipalmitoyl-sn-glycero-3-phosphocholine (DPPC) and 1,2-dioctadecanoyl-sn-glycero-3-phosphocholine (DSPC), along with DSPE-GPLGVRGK-mPEG_2K_, for the mitochondrial-targeted co-delivery of doxorubicin (DOX) and epigallocatechin-3-gallate (EGCG). EGCG targets mitochondria to enhance the efficacy of DOX therapy by influencing the function of the mitochondrial electron transport chain (mETC) and alleviating tumor fibrosis, thereby improving immunotherapy outcomes in triple-negative breast cancer (TNBC) (Figure 1). First, surface modification with 1,2-distearoyl-sn-glycero-3-phosphoethanolamine-N-poly (ethylene glycol) 2000 (DSPE-PEG_2K_) enhances the water dispersibility and biocompatibility of the nanoparticles while prolonging the blood circulation time of EGCG and DOX. After accumulation in tumor tissues via the enhanced permeability and retention effect, the outer shell of PEG is removed by matrix metalloproteinase-2 (MMP-2) overexpression in the tumor microenvironment, thereby exposing phospholipid bilayers and facilitating cellular internalization. Subsequently, the nanovesicles are extensively internalized by tumor cells, leading to the rapid release of EGCG and DOX within the intracellular tumor compartment. EGCG targets mitochondria and functions in conjunction with DOX to generate ROS that damage mitochondria, significantly enhancing DOX-induced ICD, and promoting cytotoxic T lymphocyte activation and tumor infiltration. Concurrently, EGCG is rapidly released from nanovesicles into tumor tissues through membrane fusion, alleviating dense tumor fibrosis and overcoming barriers to T-cell infiltration. This ultimately boosts the antitumor immunity and significantly enhances the therapeutic efficacy of these drugs. This study demonstrated that EGCG amplifies the ICD effect of DOX by inducing mitochondrial oxidative stress and overcoming the physical barriers that hinder T-cell infiltration, thereby enhancing immunotherapy for TNBC. These findings may pave the way for the development of novel cancer immunotherapy strategies. Furthermore, our study expands the application of EGCG in cancer immunotherapy and uncovers novel mechanisms underlying its antitumor activity.

## 2 Materials and methods

### 2.1 Materials

DSPE-mPEG_2K_ was acquired from Ponsure Co. (Shanghai, China). EGCG was purchased from Shanghai Yuanye Technology Co. Ltd. (Shanghai, China). mPEG_2K_-NHS, Fmoc-GPLGVRGK (Gly-Pro-Leu-Gly-Val-Arg-Gly-Lys) peptide, and DSPE-NHS were obtained from Xi’an Ruixi Company. MMP-2, gallic acid (GA), and Folin-Ciocalteu reagent were obtained from Sigma Chemical Co. CCK-8 was purchased from Dojindo (Kumamoto, Japan). MitoTracker Green FM was purchased from Invitrogen (Carlsbad, CA). ATP Assay Kit, Glucose Colorimetric Assay Kit (GOD/POD Method), DCFH-DA, MitoSO™ Red, and Hoechst were purchased from Beyotime. A Mitochondrial Complex Activity Assay Kit was purchased from Absin. The following antibodies for IHC-Fr were purchased from Abcam: [EPR24331-53] (ab270993, 1:100 dilution), Goat Anti-Rabbit IgG H&L (shown in red), anti-CD3 antibody (ab237721, 1:500 dilution), anti-Calreticulin antibody [EPR3924] - ER Marker (ab92516), anti-CD4 antibody [EPR19514] (ab183685, 1:200 dilution), and anti-CD8 alpha antibody [EPR21769] (ab217344, 1:500 dilution). For flow cytometry, the following antibodies were purchased from BD Biosciences (Shanghai): Anti-CD11c-FITC (557,400), anti-CD86-APC (558,703), anti-CD45-APC (559,864), anti-CD8-PE (553,033), anti-CD4-FITC (553,046), anti-interferon-γ-FITC (anti-IFN-γ-FITC, 554,411), and anti-Foxp3-PE (560,414) (all at a 1:100 dilution). Anti-CD25-APC (17-0257-42) for flow cytometry (1:100 dilution) was purchased from Invitrogen. Anti-Calreticulin antibody [EPR3924] - ER Marker (ab92516) for IF (1:300) and anti-HMGB1 antibody (ab18256) for IF (1 μg/mL) were purchased from Abcam (UK). Other reagents were obtained from Aladdin Reagent Company (China).

### 2.2 Cell lines and animals

For *in vitro* experiments, the mouse breast cancer cell line 4T1 and the fibroblast cell line NIH3T3 were obtained from Shanghai Bogoo Biotechnology Co., Ltd. NIH3T3 cells were cultured in Dulbecco’s Modified Eagle Medium supplemented with 10% fetal bovine serum (FBS), 2 mM L-glutamine, and 0.1 mM nonessential amino acids and maintained at 37°C in a humidified atmosphere containing 5% CO_2_. 4T1 cells were incubated in Roswell Park Memorial Institute (RPMI) 1640 medium enriched with 10% FBS and 2.5 g/L glucose and maintained at 37°C in a standard environment. Cells in the exponential growth phase were used for subsequent experiments.

For *in vivo* experiments, female BALB/c mice (18–20 g, 6–8 weeks old) were obtained from Hangzhou Medical College (Hangzhou, China). The mice were housed under pathogen-free conditions and maintained at 25°C with controlled humidity and without food or water limitations. This study was approved by the Animal Policy and Welfare Committee of Hangzhou Medical College (Animal Use Permit number: SYXK [Zhe] 2019-0011).

### 2.3 Synthesis and characterization of DSPE-GPLGVRGK-mPEG_2K_

Approximately 100 mg of mPEG_2K_-NHS was added to 3 mL N,N-dimethylformamide (DMF). Fmoc-GPLGVRGK peptide (1.0 equivalent) and triethylamine (3.0 equivalent) were then added, and the mixture was dissolved thoroughly. The reaction was performed at room temperature for 12 h. Piperidine (0.6 mL) was added and the mixture was allowed to react for an additional 30 min. The solvent was then removed under reduced pressure. The resulting product was then redissolved in an appropriate volume of DMF and transferred to a large volume of ice-cold ethyl ether to induce precipitation of the product. The precipitate was collected by filtration to obtain NH_2_-GPLGVRGK-mPEG_2K_. A total of 100 mg of NH_2_-GPLGVRGK-mPEG_2K_ was added to DMF (3 mL). DSPE-NHS (0.9 equivalent), and triethylamine (3.0 equivalent) were added and dissolved completely in the solution. The reaction proceeded at room temperature for 12 h. The reaction solution was then transferred to a dialysis bag and dialyzed with pure water for 24 h. The dialysis solution was collected and freeze-dried to obtain the product (DSPE-GPLGVRGK-mPEG_2K_). The structures of the products were studied using ^1^H NMR spectroscopy.

### 2.4 Synthesis of dual-drug-loaded nanovesicles (EEDNV) and characterization of EEDNV

Drug-loaded nanovesicles were prepared using a thin-film hydration method (Wu et al., 2019). To prepare EENV, DPPC, DSPC, DSPE-GPLGVRGK-mPEG_2K_, and EGCG, the compounds were dissolved in a solvent mixture of chloroform and methanol. The mixture was vacuum-dried to obtain a thin film, which was subsequently rehydrated in phosphate-buffered saline (PBS) at 50°C and squeezed through polycarbonate filters to prepare nanovesicles (EENV). To load DOX into the hydrophilic core of the EENV for the preparation of the EEDNV, the lipid film was hydrated with a 300 mM ammonium sulfate solution. The microvesicles were dialyzed against PBS (pH 7.4) for 24 h to establish an ammonium sulfate gradient after extrusion. Free DOX (with a DOX-to-EGCG mass ratio of 10%) was subsequently added to the nanovesicles and hydrated for 20 min at 55°C. Next, the nanovesicles (EEDNV) were dialyzed against PBS (pH 7.4) to remove the free DOX. Meanwhile, PNV (enzyme-insensitive nanovesicles, which replaced DSPE-GPLGVRGK-mPEG_2K_ with DSPE-mPEG_2K_), ENV (EEDNV without EGCG and DOX), EDNV (EEDNV without EGCG), and enzyme-insensitive corresponding controls of EENV, EDNV and EEDNV (PENV, PDNV, and PEDNV) were prepared using the same method. Dynamic light scattering (DLS, Zetasizer, UK) and transmission electron microscope (TEM, Talos L120C, USA) were used to determine the size distribution and morphology of the samples. The encapsulation efficiency and loading ratio of EGCG and DOX were determined using a multi-detection microplate reader (BioTek, USA).

### 2.5 De-PEGylation of EEDNV

EEDNV was incubated with various concentrations of matrix metalloproteinase-2 (MMP-2) (0, 25, 250, and 500 μg/mL). The changes in size and polydispersity index (PDI) of EEDNV were measured at incubation times of 0, 10, 20, 30, 40, 50, 60, 90, and 150 min using DLS.

### 2.6 Colloid stability of EEDNV

To assess the serum stability of the dual drug-loaded nanovesicles, EEDNV was incubated in PBS containing 10% FBS at 37°C. The size of the EEDNV was monitored at 1, 2, 4, 6, 8, 12, and 24 h using DLS. For long-term stability evaluation, EEDNV in PBS with 10% FBS was stored at 4°C, and the size of EEDNV was measured at 1, 2, 3, 4, 5, and 6 days using DLS.

### 2.7 Drug release

Briefly, 0.5 mg/mL of EENV and PENV were incubated with or without 40 μg/mL of MMP-2 at 37°C. At various time points, samples were collected and centrifuged for 5 min in 100 kDa ultrafiltration tubes. The EGCG content in the supernatant was determined using the Folin-Ciocalteau method, which was adapted for use with a microplate reader. To minimize the oxidation of EGCG, nitrogen was introduced into the water or test tube to displace the oxygen before the experiment.

To investigate the release of DOX from the nanovesicles, 3 mL of EDNV, EEDNV, and PEDNV suspensions (100 μg/mL DOX) containing 40 μg/mL MMP-2 was placed in dialysis tubes with a molecular weight cut-off of 3500 Da. In addition, 10 mL of 40 μg/mL MMP-2 was added to the exterior of each dialysis bag. Samples were collected at various time points, ranging from 0 to 24 h. The release of DOX from the nanovesicles into the solution was measured using a multi-detection microplate reader (BioTek, USA).

### 2.8 Cell viability

The CCK-8 assay was used to detect the cytotoxicity of the samples. 4T1 tumor cells were incubated for 24 h. ENV and PNV, with or without MMP-2, were then added to the cells at 1, 10, 50, 100, and 500 μg/mL for 24 h. To avoid potential confounding factors that would influence the outcome of the experiments, in the enzyme-activated groups, the nanovesicles were treated with 40 μg/mL MMP-2 for 60 min in MMP-2 groups in this experiment and all subsequent experiments. To determine the cytotoxicity of the drug-loaded samples, 4T1 cells were incubated for 24 h and subsequently treated with various concentrations of EDNV, EENV, or EEDNV, with or without MMP-2, for an additional 24 h. The cells were then analyzed using the CCK-8 assay.

### 2.9 Cellular uptake and co-localization experiments

To observe the cellular uptake and drug distribution of the nanovesicles *in vitro*, 4T1 cells were incubated in culture dishes (3 × 10^4^ cells/well) for 24 h. The cells were then cultured with EDNV + MMP-2, and EEDNV with or without MMP-2 at a consistent DOX concentration of 2 μg/mL for 12 h. After incubation, cells were washed with PBS. Subsequently, 100 nM green fluorescent MitoTracker was added, and the nuclei were stained with Hoechst 33,342 before visualization under a fluorescence microscope.

### 2.10 Assay of mitochondrial complexes activity, glucose level, and ATP level

To clarify the impact of the nanovesicles on the activities of mitochondrial complexes I and II, 0.1 mg/mL of EEDNV and EENV, along with MMP-2, were used to treat cells. After 12 h of incubation, the activities of the mitochondrial complexes were measured according to the manufacturer’s instructions.

The cells were seeded in 6-well plates for 12 h and then co-incubated with 100 μg/mL of EEDNV and EENV with MMP-2. After 12 h, glucose levels were measured according to the protocol.

The 4T1 cells were seeded and incubated for 24 h. Subsequently, 100 μg/mL of EEDNV and EENV, along with MMP-2, was added and cultured for an additional 12 h. Subsequently, the cells were treated with an ATP assay kit, and bioluminescence was measured using a luminometer (Spark 10 M, Tecan).

### 2.11 Cell respiration study

In this experiment, 4T1 cells were seeded in dishes and co-incubated with PBS, EENV (100 μg/mL), and EEDNV (100 μg/mL) under normoxic conditions. The basic RPMI-1640 medium was used as a control. After 12 h, the cells were trypsinized and diluted in 10 mL of fresh RPMI-1640 medium. Subsequently, the oxygen (O_2_) levels in each chamber were measured using an oxygen-dissolving meter (JPSJ-605F, Shanghai).

### 2.12 Assessment of intracellular ROS levels

4T1 cells were seeded in pre-prepared dishes at a density of 5 × 10^4^ cells/well and cultured for 1 day. Subsequently, the cells were treated under various conditions for 12 h with a consistent DOX concentration of 1.2 μg/mL and EGCG dose of 16 μg/mL. The cells were then incubated with DCFH-DA for 30 min and washed with a culture medium lacking FBS to eliminate any unbound DCFH-DA. Imaging was performed using confocal laser scanning microscope (CLSM), and the fluorescence intensity was quantified using the ImageJ software.

### 2.13 Immunogenic cell death *in vitro*

The ICD effect of EEDNV was detected by analyzing CRT exposure and nuclear HMGB1 efflux in 4T1 cells *in vitro*. 4T1 cells were treated with PBS, EEDNV + MMP-2 and PEDNV + MMP-2 at an identical DOX concentration of 1.6 μg/mL for 12 h (n = 3). Afterward, the cells were stained with an anti-CRT primary antibody (Abcam, ab92516, 1:300 for IF) and Alexa488-conjugated secondary antibody (Abcam, ab150077, 1:500) for 30 min. The cells were then analyzed by CLSM. For detecting HMGB1 efflux by CLSM, the cells were permeabilized in 0.1% Triton X-100 for 5 min and then blocked in 5% FBS for 1 h at room temperature before staining with anti-HMGB1 antibody (Abcam, ab18256, 1 μg/mL).

### 2.14 Second harmonic generation imaging (SHGI), immunohistochemistry (IHC) staining of fibrosis *ex vivo*, and distribution of EENDV *in vivo*

A TNBC mouse model was established by subcutaneously injecting 5 × 10^6^ 4T1 cells and 5 × 10^6^ NIH3T3 cells into the right fat pad of the mice. After several days, the mice were used for subsequent experiments when the tumor volume reached approximately 200 mm^3^.

To assess whether EEDNV alleviated tumor fibrosis *in vivo*, the mice were divided into six groups (n = 3). The mice were injected via the tail vein with PBS, 100 mg/kg ENV, 16 mg/kg EGCG combined with 1.2 mg/kg DOX, EENV, EDNV, and EEDNV (#1 PBS, #2 ENV, #3 DOX + EGCG, #4 EENV, #5 EDNV, and #6 EEDNV), all at identical concentrations of DOX (1.2 mg/kg) and EGCG (16 mg/kg) once every other day for 1 week. After 24 h following the last administration, the mice were euthanized and the samples were harvested. SHG microscopy was used to evaluate the characteristics of collagen fibers in the tumor tissue (Jia et al., 2023). Fluorescence images were captured using an AniView ex-imaging system (BLT, China) to assess the distribution of EEDNV in the tissues. Moreover, to determine whether the nanovesicles promoted lymphocyte infiltration by inhibiting tumor fibrosis, immunofluorescence (IF) staining with an anti-CD3 antibody was performed on the tumor samples (n = 3).

### 2.15 Antitumor performance in a TNBC mouse model

The TNBC mouse model was established as described previously. Once the tumor volume reached approximately 100 mm^3^, mice were used for subsequent experiments.

For the efficacy experiments, tumor-bearing mice were divided into five groups (n = 5) and treated as follows: #1 PBS, #2 ENV, #3 DOX + EGCG, #4 EENV, #5 EDNV, and #6 EEDNV. All groups received identical doses of DOX and EGCG at 1.2 and 16 mg/kg, respectively. The various formulations were intravenously administered once every 3 days for 3 weeks. Body weight and tumor volume were measured every 3 days. The tumor tissues were collected, weighed, and photographed. The tumors and primary organs were stained with hematoxylin and eosin (H&E).

For the assessment of mitochondrial superoxide, the model mice were divided into six groups (n = 3) and treated with the following: #1 PBS, #2 ENV, #3 DOX + EGCG, #4 EENV, #5 EDNV, and #6 EEDNV, all at identical doses of DOX and EGCG dose of 1.2 and 16 mg/kg, respectively. Different formulations were administered intravenously once every 3 days for a total of four times. After 24 h after the last tail vein injection, 20 μL of 1 mM MitoSO™ Red was administered intratumorally. After an additional 12 h, the tumors were collected, cryosectioned, and stained with Hoechst 33,342. Tissue sections were observed and photographed using CLSM at excitation and emission wavelengths of 396 and 610 nm, respectively.

For immunoassays, tumor-bearing mice were divided into six groups and treated as follows: #1 PBS, #2 ENV, #3 DOX + EGCG, #4 EENV, #5 EDNV, and #6 EEDNV. All groups received identical doses of DOX and EGCG at 1.2 and 16 mg/kg, respectively. Different formulations were administered intravenously once every 3 days for a total of four doses. The mice were euthanized on the third day after the last administration. Tumor samples were immunostained using an anti-CRT primary antibody (ab92516) and analyzed using CLSM (n = 3). For the analysis of DC maturation, lymph nodes were collected, homogenized into a suspension, stained with anti-CD11c-FITC and anti-CD80-PE, and subsequently analyzed using flow cytometry. To identify tumor-infiltrating regulatory T cells (Tregs) and IFN-γ^+^CD8^+^ T cells, T lymphocytes were stained with anti-CD45-APC and anti-CD25-APC according to established protocols. The cells were analyzed using flow cytometry (n = 3). IF staining was performed to visualize the expression of CD4^+^ and CD8^+^ T cells in TNBC tumors.

### 2.16 Statistics

All data are presented as mean ± standard deviation (SD). GraphPad software was used for statistical analyses. A *t*-test was used to determine the differences between the two groups.

## 3 Results

### 3.1 Synthesis and physicochemical properties of EEDNV

To achieve mitochondrion-targeted co-delivery of EGCG and DOX to tumor cells, an MMP-2-sensitive nanovesicle was developed by integrating EGCG and DOX into a nanoplatform. Initially, a PEG chain was covalently conjugated to the DSPE via a peptide spacer (GPLGVRGK) through a series of condensation reactions (Supplementary Figure S1). The resulting DSPE-GPLGVRGK-mPEG_2K_ was characterized using ^1^H NMR spectroscopy. The ^1^H NMR spectrum of Fmoc-GPLGVRGK exhibited peaks ranging from 7.25 to 8.25 ppm (peaks in box 1), 4.5 to 3.5 ppm (peaks in box 2), and 2.5 to 1.5 ppm (peaks in box 3), corresponding to the hydrogen proton peak of the benzene ring, -N-C*H*-, -N-C*H*_*2*_-C=O, and -CH-C*H*_*2*_-CH_2_-, respectively. The characteristic peaks in the ^1^H NMR spectrum of DSPE-GPLGVRGK-mPEG_2K_ were as follows: 3.53 ppm (-O-C*H*_*2*_-CH_2_-O-, PEG chain) and 1.24 ppm (-C*H*_*2*_-C*H*_*2*_-C*H*_*2*_-, hydrophobic DSPE chain). The disappearance of the peaks in box 1, along with the emergence of two new peaks corresponding to the PEG and DSPE chains, indicates the successful conjugation of DSPE-GPLGVRGK-mPEG_2K_ (Supplementary Figure S2).

**FIGURE 2:**
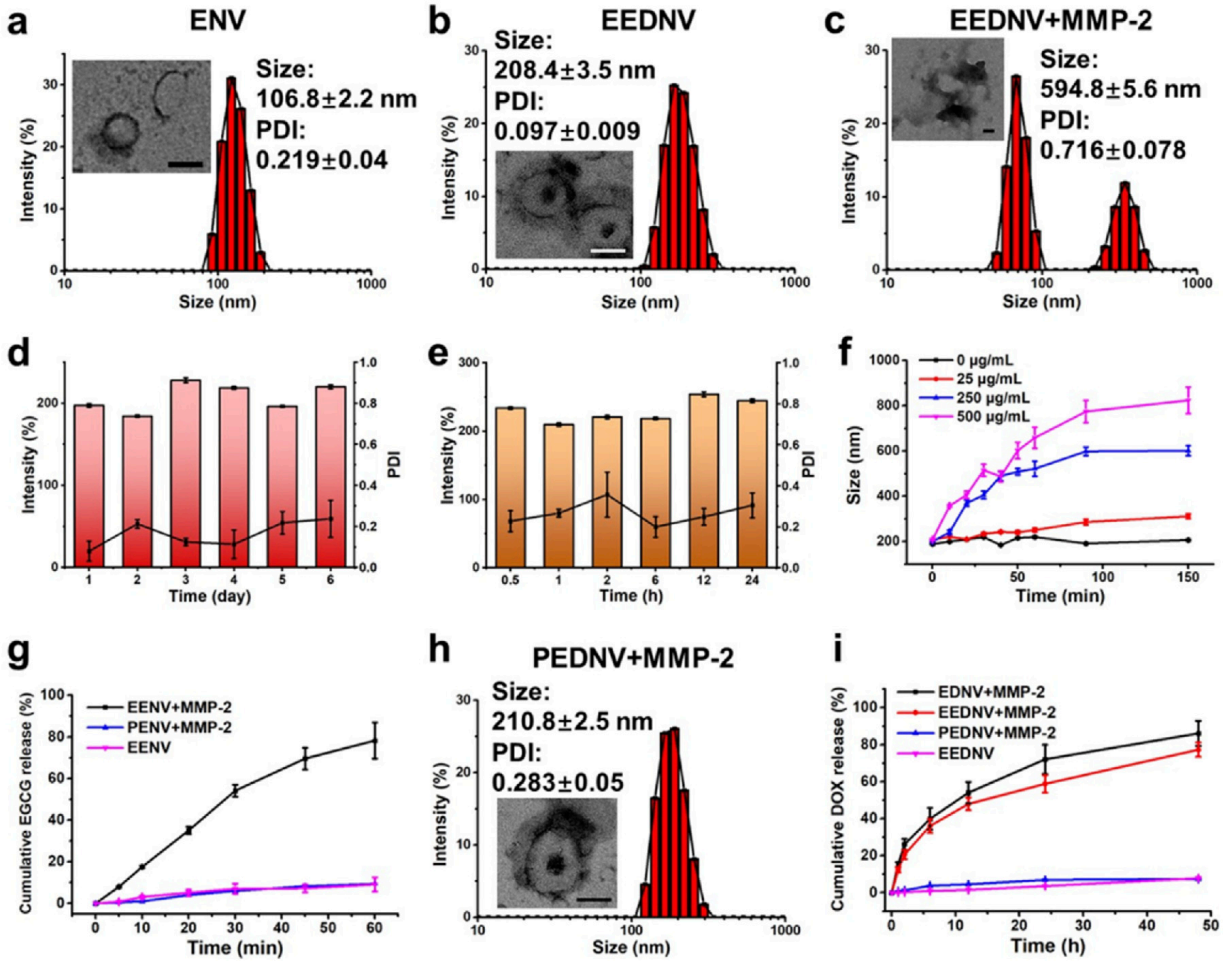
Characterization of physicochemical properties of nanovesicles *in vitro*. **(a–c)** Hydrodynamic diameters and typical TEM images of ENV **(a)**, EEDNV **(b)**, and EEDNV nanovesicles cultured with MMP-2 **(c)**. **(d,e)** DLS-determined long-term stability **(d)** and serum stability **(e)** of EEDNV. **(f)** Changes in EEDNV size after incubation with MMP-2 for different durations. **(g,i)** Cumulative drug release profiles of PENV, EENV **(g)**, EDNV, EEDNV, and PEDNV **(i)** after different treatments. **(h)** Representative TEM images of the PEDNV nanovesicles incubated with MMP-2. All data are expressed as the mean ± SD, *n* = 3.

Subsequently, dual-drug-loaded enzyme-sensitive and enzyme-insensitive nanovesicles, blank nanovesicles, and various single-drug-loaded control nanovesicles were prepared using a modified thin-film hydration method (Yang et al., 2022). DLS and TEM revealed that the enzyme-sensitive blank nanovesicles had an average particle size of approximately 106.8 ± 2.2 nm, and exhibited a hollow spherical morphology (Figure 2a). The hydrated particle size and PDI of the MMP-2-insensitive blank nanovesicles were 86.0 ± 0.3 nm and 0.089 ± 0.01, respectively (Supplementary Figure S3a). EGCG can be encapsulated in lipid bilayers through interactions with the neutral phospholipid DPPC. EEDNV was constructed by encapsulating DOX inside EENV using the transmembrane ammonium sulfate gradient method. The DLS and TEM results confirmed the homogeneity of EEDNV, which had a hydrated particle size of 208.4 ± 3.5 nm and demonstrated excellent stability (Figures 2b,d,e). The zeta potential results of the various nanovesicles indicated the successful drug encapsulation of EGCG and DOX (Supplementary Table S1). The DLS results for the remaining nanovesicles confirmed their successful synthesis (Supplementary Figure S3). The encapsulation efficiency of EGCG and DOX was 96.1% ± 0.40% and 69.1% ± 1.3%, respectively, as measured by the Folin-Ciocalteau method and UV-Vis spectrophotometry (Supplementary Table S2). EGCG interacts with both the hydrophobic and hydrophilic regions of the phospholipid bilayer. The core mechanism underlying the increased particle size of nanovesicles after drug loading is attributed to physical expansion caused by drug-membrane interactions and apparent aggregation induced by surface charge alterations. To demonstrate enzyme responsiveness, EEDNV were co-incubated with 40 μg/mL MMP-2 for 4 h, and the dissociation of the spatial structure of EEDNV induced by de-PEGylation was observed using TEM (Figure 2c). Furthermore, the rate of vesicle dissociation increased with increasing MMP-2 concentration and incubation time (Figure 2f; Supplementary Figure S4). However, the size and morphology of PEDNV remained largely unchanged in the presence of MMP-2 (Figure 2h).

The cumulative release of EGCG and DOX from nanovesicles under various conditions was also investigated. The results indicated that drug release could be effectively controlled, further demonstrating the enzymatic responsiveness of the nanovesicles (Figures 2g,i). EGCG was rapidly released from EENV in the presence of MMP-2, with the drug release rate reaching approximately 80.0% within 1 hour. In contrast, EGCG release was significantly inhibited in the absence of MMP-2 (Figure 2g). PENV exhibited minimal release of EGCG. The release pattern of DOX mirrored that of EGCG; however, DOX was released more slowly because of its encapsulation within the internal hydrophilic core of the nanovesicles (Figure 2i). The findings revealed that EEDNV remained stable in the bloodstream and rapidly released EGCG to alleviate tumor fibrosis, thereby enhancing the accumulation of both EGCG and DOX in tumor cells upon reaching the tumor tissue.

### 3.2 Mitochondrial targeting and cytotoxicity of EEDNV

**FIGURE 3:**
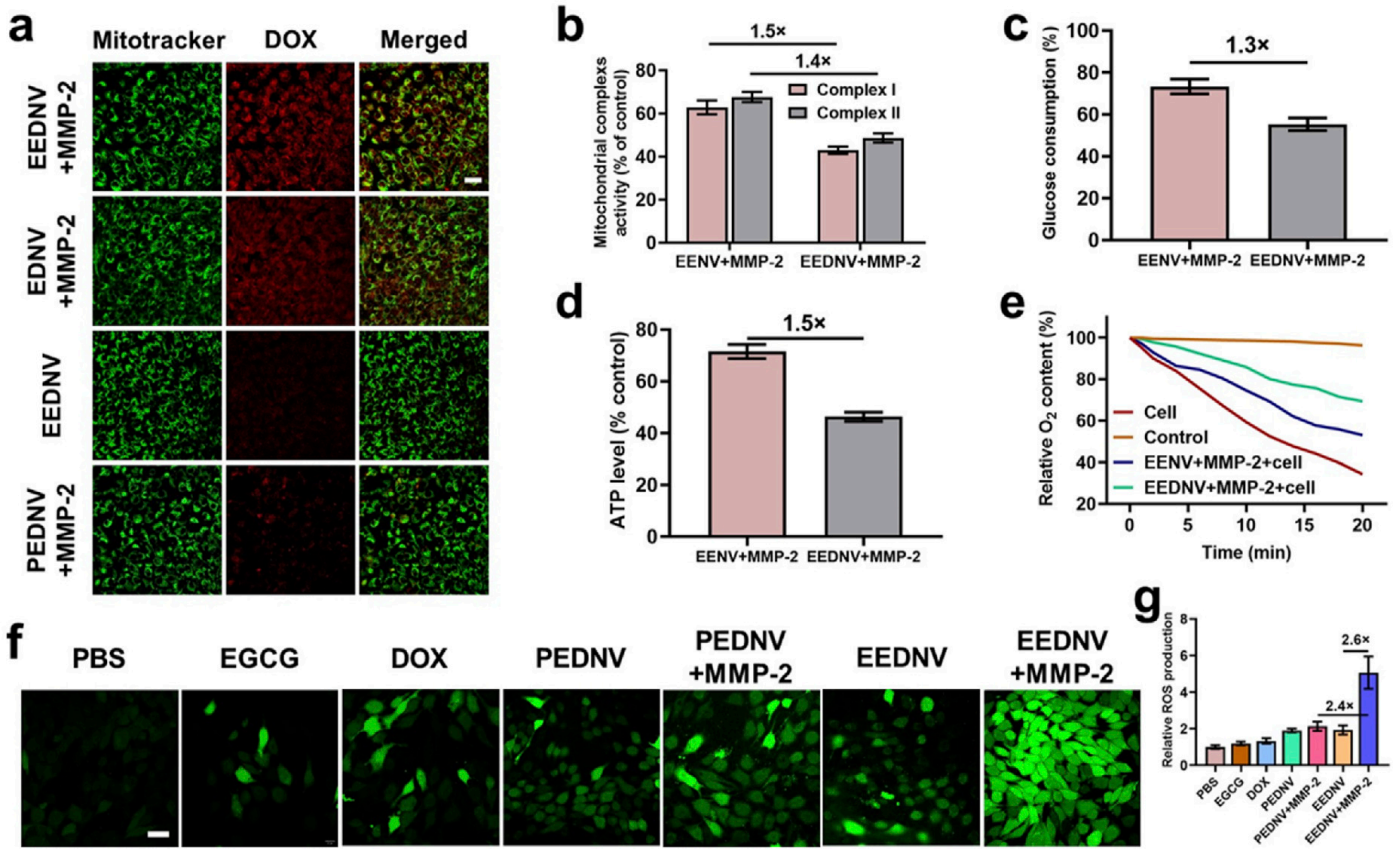
Contribution of EENV and EEDNV to mETC disruption and ROS generation. **(a)** Confocal fluorescence co-localization of mitochondria and nanovesicles with or without MMP-2. Scale bar = 50 μm. **(b)** The activities of mitochondrial complexes I and II in 4T1 cells after treatment with 100 μg/mL of EENV and EEDNV for 12 h. **(c)** Glucose consumption of 4T1 cells after treatment. **(d)** Intracellular ATP level of 4T1 cells after treatment. **(e)** Relative O_2_ content in the medium. O_2_ content in the blank medium served as a control. **(f)** Fluorescence images of 4T1 cells stained with 10 μM DCFH-DA. Scale bars = 20 μm. **(g)** Relative ROS production. Error bars represent the mean ± SD, *n* = 3.

Given the successful preparation of the EEDNV, mitochondrial targeting was validated using CLSM. Previous studies have shown that EGCG targets mitochondria and disrupts the mitochondrial mETC (Liu et al., 2023). The degree of colocalization between the DOX-loaded nanovesicles and mitochondria was assessed using Pearson’s colocalization coefficient. The results indicated that DOX in EEDNV exhibited a high degree of mitochondrial colocalization, with a colocalization coefficient of 0.89 ± 0.03. This was significantly higher than that observed in EDNV, which had an average Pearson coefficient of 0.43 ± 0.01 (Figure 3a). Mitochondria-targeted DOX induces severe mitochondrial dysfunction by converting it to semiquinone radicals, leading to ROS generation. This mechanism serves as a non-Fenton chemodynamic therapy (CDT) and may help circumvent chemoresistance (Jana and Zhao, 2022; Zhou M.-X. et al., 2023). Thus, the results suggest that EGCG-mediated mitochondrial-targeted nanovesicles can effectively deliver DOX to the mitochondria and that EGCG synergizes with DOX to enhance the CDT. To mitigate the effect of dePEGylation on the uptake process, enzyme-sensitive nanovesicles were incubated overnight *in vitro* with or without MMP-2. These findings indicated that PEG in the lipid layer significantly obstructed cellular drug uptake. DePEGylation can enhance the tumor-targeting ability and cellular uptake of enzyme-sensitive nanovesicles, demonstrating the importance of designing tumor microenvironment-responsive nanovesicles (Figure 3a). The cell uptake was then quantified using fluorescence of DOX, and the fluorescence quantification was done with ImageJ. The results showed that the fluorescence intensity was not significantly different between the EDNV + MMP-2 and EEDNV + MMP-2 two groups, because the 4T1 cells were co-incubated with EDNV + MMP-2 and EEDNV + MMP-2 containing the same concentration of DOX. EGCG-mediated mitochondria-targeted DOX does not affect its cellular uptake behaviors. The fluorescent intensity of DOX in the PEDNV + MMP-2 group was very weak, which was reduced by around 3-fold compared with the EEDNV + MMP-2 group (Supplementary Figure S5). These results suggest that PEG in the lipid layer hindered cellular uptake of the nanovesicles.

We subsequently investigated the effects of EENV and EEDNV on the function of mETC by assessing the activity of mitochondrial complexes I and II (Fontana et al., 2020). Under the specified conditions, we observed a significant decrease in the activity of mitochondrial complex I, which reduced to 62.8% and 42.9% after 12 h of co-incubation of 4T1 cells with EENV and EEDNV, respectively, and complex II also experienced a decline of 67.6% and 48.6%, respectively (Figure 3b). These results indicate that EGCG and DOX synergistically reduced the activity of complexes I and II. Both EENV and EEDNV are capable of perturbing the mETC, thereby influencing the activity of these complexes. This disruption of the mETC is expected to diminish the demand for glucose during oxidative phosphorylation and reduce the oxygen consumption for mitochondrial respiration within cells (Siedlar et al., 2023). Our experimental results also demonstrated that glucose consumption was reduced and ATP levels were diminished in cells treated with EENV or EEDNV (Figures 3c,d). Notably, EEDNV appeared to inhibit glucose consumption and ATP levels to a greater extent than EENV. Additionally, decreased oxygen demand was confirmed by measuring the oxygen content in the culture medium containing 4T1 cells after treatment. Compared with the control group, oxygen consumption in both the EENV- and EEDNV-treated groups was significantly slower (Figure 3e).

DOX can accept electrons to form DOX semiquinone, which subsequently transfers electrons to O_2_ by quinone one-electron redox cycling to generate ROS (Nagakubo et al., 2019; Smith et al., 2020). However, disruption of mETC by EGCG may promote electron leakage, leading to the formation of additional DOX semiquinones. The O_2_ retained due to respiration inhibition can then accept electrons to produce more ROS, further amplifying the efficacy of CDT. Using a fluorescent ROS probe, both EGCG and DOX were found to slightly enhance cellular ROS production (Figure 3f). Cells treated with EEDNV + MMP-2 exhibited significantly higher ROS levels, which was attributed to the high uptake rate of enzyme-sensitive nanovesicles. Furthermore, due to the biological challenges associated with poor mitochondrial targeting by DOX, it alone only slightly increased the ROS levels. The results showed that the green fluorescence intensity was not significantly different between the PEDNV and PEDNV + MMP-2 two groups because the inadequate cellular uptake of both PEDNV and PEDNV + MMP-2 groups led them the production of less ROS (Figures 3f,g). EEDNV demonstrated superior cellular ROS production compared with EGCG, suggesting a potential interaction between DOX and EGCG. After the addition of EGCG, the electron capture effect of DOX was enhanced, resulting in an increased production of ROS. Furthermore, subsequent animal experiments demonstrated that ROS generation was primarily mediated by the superoxide anion (·O_2_^−^) (Supplementary Figure S10). It is well established that ROS may contribute to ICD-associated immunogenicity and strategies that induce ROS such as photodynamic therapy radiation therapy and CDT may enhance the effectiveness of ICD (Guo et al., 2021). Therefore, these findings also suggest that the dual drug-loaded nanovesicle-augmented CDT effect can ablate tumors, induce ICD, and recruit tumor-infiltrating lymphocytes that are significantly enriched in peritumoral tissues. Next, we explored the capacity of EEDNV to elicit ICD by assessing the surface expression of calreticulin (CRT) and the efflux of high mobility group protein B1 (HMGB1) from the nucleus *in vitro*. There was virtually no CRT signal present in the PBS and PEDNV + MMP-2 groups, while the EEDNV + MMP-2 groups significantly enhanced CRT expression on the surface of the tumor cells (Supplementary Figure S6), suggesting that enzyme-sensitive nanovesicles can trigger apoptosis and promote CRT exposure on the membranes of 4T1 cells. In a similar result, the intracellular fluorescence of HMGB1 was observed to be 5.5 times lower in the EEDNV + MMP-2 group compared to the PBS group (Supplementary Figure S6c).

Additionally, the results were validated using cytotoxicity experiments. All the blank nanovesicles exhibited excellent biosafety (Supplementary Figure S7a). DePEGylation improved DOX chemotherapy, whereas EGCG synergistically enhanced the CDT of DOX (Supplementary Figure S7b–d).

### 3.3 EEDNV-mediated increase in tumor-infiltrating immune cells

**FIGURE 4:**
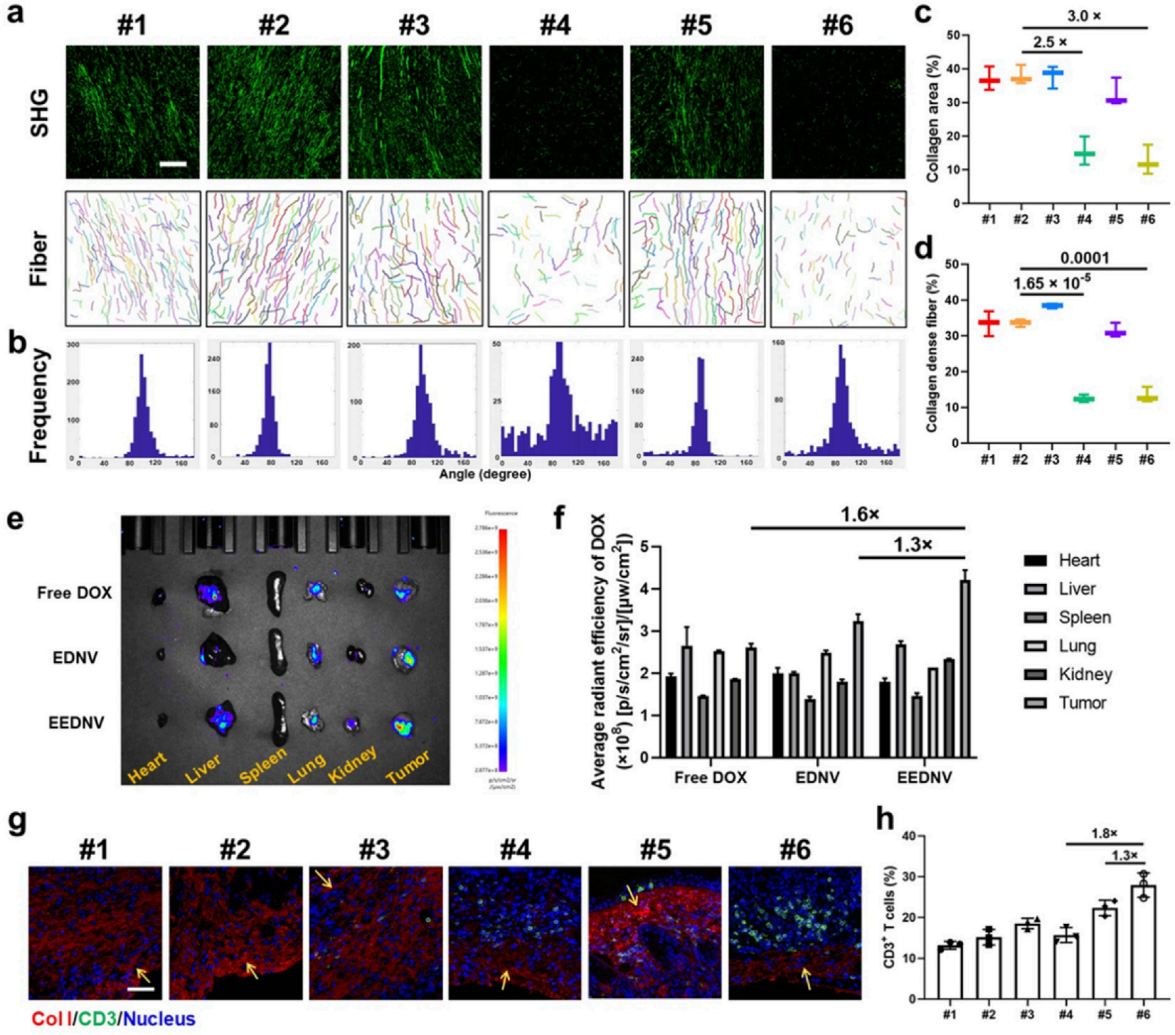
EEDNV relieves tumor fibrosis and facilitates the delivery of chemotherapeutic agents to tumors, resulting in increased lymphocyte infiltration. **(a)** Representative SHG and separate collagen fiber images showing the collagen fiber framework of 4T1/NIH3T3 tumors after different treatments. Images were acquired using CT-FIRE software (scale bar = 100 μm). The “mean orientation/alignment” values of collagen fibers were #1 100.8/0.87; #2 75.4/0.94; #3 97.5/0.81; #4 97.0/0.32; #5 86.3/0.83; #6 90.6/0.66; respectively. (n = 3 mice) **(b)** The curve of collagen fiber distribution to compare collagen alignment. **(c,d)** Quantitative parameters, including the percentage of collagen area **(c)** and collagen-dense fibers **(d)** in **(a)**. **(e)** Typical *ex vivo* fluorescence images of the samples from mice treated with Free DOX, EDNV, or EEDNV. **(f)** Average radiant efficiency of the mice **(e)** (n = 3). **(g)** IF staining of collagen I in 4T1/NIH3T3 tumors. Scale bar = 50 μm. Yellow arrows indicate sites of immune infiltration. **(h)** Intratumoral infiltration of CD3^+^ T-cells (n = 3 mice). Error bars represent the mean ± SD.

Unfortunately, T-cell infiltration is impeded by the dense fibrotic tissue surrounding solid tumors (Riley et al., 2019). Previous studies demonstrated that EGCG could block the TGF-β/Smad signaling pathway *in vitro* and *in vivo*. WB results showed that 2 ng/mL TGF-β1 adequately activated the TGF-β/Smad pathway in NIH3T3 cells. However, EGCG treatment decreased pSmad2/3 expression by 8.5- and 5.0-fold when compared with TGF-β1-treated cells, suggesting that EGCG effectively blocked the TGF-β/Smad pathway in NIH3T3 cells. In addition, WB and immunohistochemical analysis showed that EGCG downregulated the protein expression of pSmad3 *in vivo*, whereas Smad protein content remains unchanged (Zhou et al., 2025). Moreover, EGCG downregulated LOXL2 through the TGF-β/Smad signaling pathway and efficiently prevented the crosslinking of collagen. The uncrosslinked collagen might have been degraded in the stroma by some hydrolases (Wei et al., 2017). Therefore, EGCG reduced the collagen I expression *in vivo* and finally alleviated tumor fibrosis. Another previous study indicated that nanovesicles loaded with an inhibitor of TGF-β receptor 1 (LY2157299) alleviated tumor fibrosis through the TGF-β/Smad signaling pathway and promoted the infiltration of cytotoxic T lymphocytes into tumor tissues to prevent immune evasion, thereby enhancing the efficacy of immunotherapy in TNBC and pancreatic cancer (Zhou M. et al., 2023). Consistent results were obtained through SHG imaging in animal experiments using the engineered EGCG and DOX dual drug-loaded enzyme-responsive nanovesicles (Figures 4a–d). The frequency curve of the fiber distribution revealed that the EENV and EEDNV groups exhibited the lowest frequency of oriented fibers (Figure 4b), indicating that the EGCG-loaded nanovesicles disrupted the intrinsic fiber alignment and reduced the length of the fibers. Furthermore, the percentage of collagen-dense fibers and the collagen area were significantly lower in the EGCG-loaded nanovesicles than in those without EGCG (Figures 4c,d). *Ex vivo* imaging indicated that DOX signals were primarily localized in the liver and tumor tissues (Figure 4e). Semi-quantitative analysis of fluorescence intensity demonstrated that EEDNV induced the highest accumulation of DOX in 4T1/NIH3T3 tumors (Figure 4f).

IF staining was performed to investigate lymphocyte infiltration after the reduction of tumor fibrosis. Collagen fibers, identified as Collagen Type I, are marked in red, whereas CD3^+^ T cells are indicated in green (Figure 4g). EENV, EDNV, and EEDNV increased the number of tumor-infiltrating lymphocytes to varying degrees, whereas the EEDNV group revealed a 1.3 and 1.8-times higher CD3^+^ T cell ratio (27.9 ± 3.0%) than that of the EENV and EDNV groups, respectively (Figures 4g,h). Notably, the EDNV and free EGCG + DOX groups had a slight effect on collagen I levels and moderately reduced the burden of the extracellular matrix (Supplementary Figure S8). Nevertheless, EGCG-loaded nanovesicles significantly decreased collagen I levels *in vivo*, consistent with the SHG observations, and then promoted infiltration of CD3^+^ T cells (Supplementary Figure S8; Figure 4g), proving that EEDNV significantly synergistically increased the number of tumor-infiltrating lymphocytes by simultaneously inhibiting the transforming growth factor-β (TGF-β) pathway and enhancing the ICD effect, thus providing a precondition for improving immunotherapy efficacy.

### 3.4 EEDNV potentiates effective cancer immunotherapy by improving mitochondria-mediated ICD and overcoming physical barriers

**FIGURE 5:**
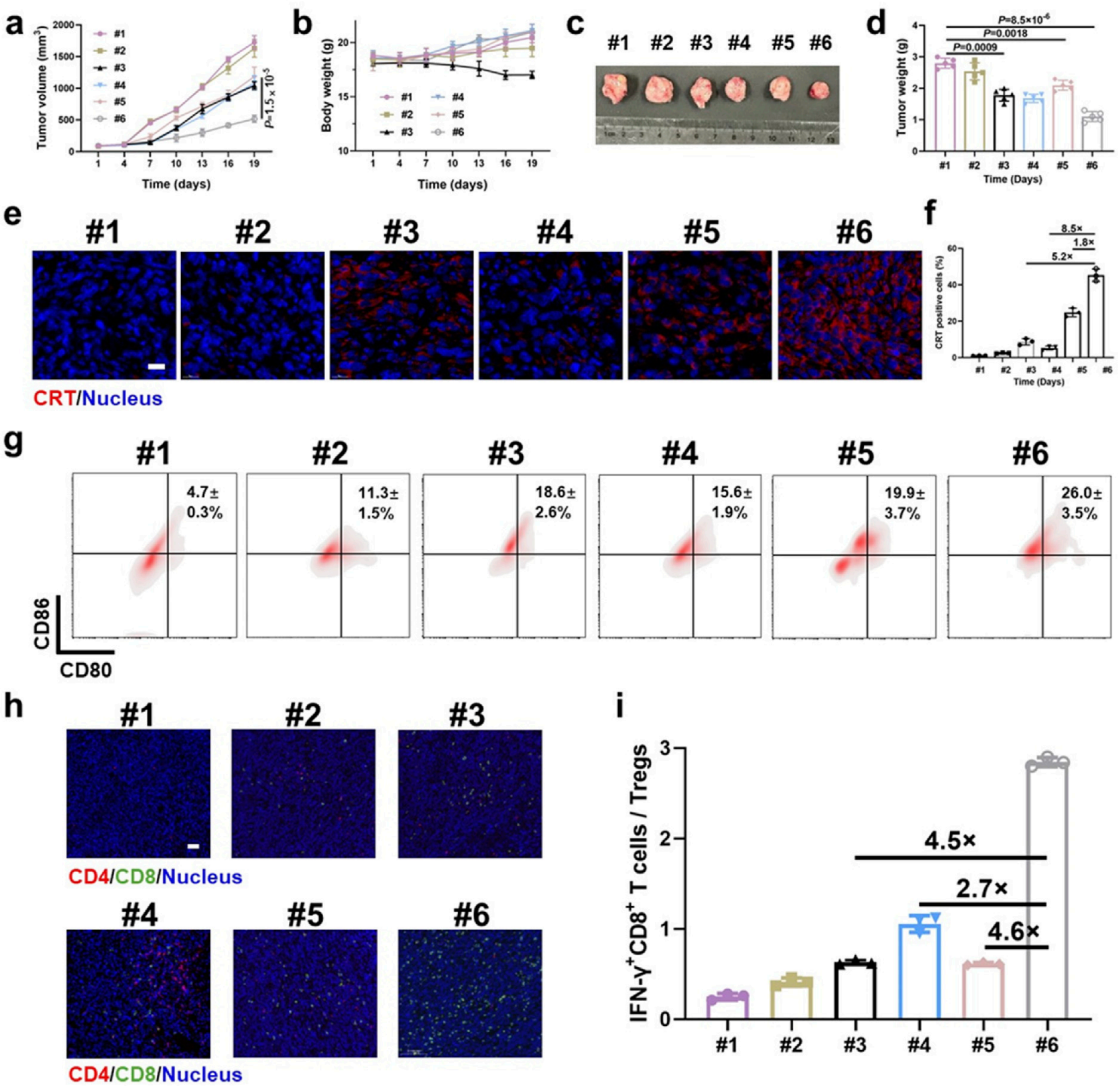
Antitumor immunotherapeutic effects of EEDNV in mice bearing subcutaneous 4T1/NIH3T3 grafts. **(a)** Tumor growth curves of TNBC mice. #1 PBS, #2 ENV, #3 DOX + EGCG, #4 EENV, #5 EDNV, and #6 EEDNV (n = 5 mice). **(b)** Average body weights of BALB/c mice after different treatments (n = 5). **(c)** Images of excised tumors after treatment (n = 5 mice). **(d)** Mean tumor weight of mice after treatments (n = 5). **(e)** CLSM examination of CRT exposure in 4T1/NIH3T3 tumors (n = 3 mice). **(f)** Fluorescence semi-quantitative analysis of CRT exposure in **(e)**. **(g)** Frequency of mature DCs in tumor-draining lymph nodes (LNs) from model mice. **(h)** IF staining of CD4^+^ and CD8^+^ T cells in 4T1/NIH3T3 tumor samples at 3 days (n = 3 mice). **(i)** Ratio of IFN-γ^+^CD8^+^ T cells to Treg cells (n = 3). Error bars represent the mean ± SD. *P-*values were derived using t-tests (two-tailed).

Because the enzyme-sensitive dePEGylation nanovesicles promoted the synergistic effects of EGCG and DOX, the experimental findings demonstrated that the group treated with EEDNV exhibited the highest efficacy among all the treatment approaches at the 19-day time point (Figures 5a,c,d). This indicated that EGCG enhanced chemo/chemodynamic therapy with DOX and significantly activated the antitumor immune response. Body weight measurements and H&E results confirmed the low toxicity of nanovesicle treatment, except for the EGCG + DOX group, which showed the slowest weight gain in mice (Figure 5b; Supplementary Figure S9). Moreover, mechanistic studies have suggested that EGCG may target the mitochondria to enhance the generation of mitochondrial ROS in synergy with DOX *in vivo*. The MitoSOX™ Red fluorescence in the EEDNV group was 5.0 times greater than that in the EDNV group (Supplementary Figure S10). The results further demonstrated that EGCG improved the mitochondrial targeting of DOX and disrupted the mETC, facilitating the formation of a substantial amount of semiquinone radicals, which ultimately induced mitochondrial oxidative stress and enhanced mitochondrial apoptosis. Furthermore, mitochondrial ROS may promote efficient mitochondrial-mediated ICD, leading to tumor cells expressing CRT on their surface, which attracts APCs. The percentage of CRT-positive cells in the EEDNV group was 1.8-fold higher than that in the EDNV group (Figures 5e,f). Our study revealed that EGCG significantly enhanced the immunogenicity of whole tumor cells induced by DOX.

Next, we assessed ICD-induced immune responses by evaluating DC maturation in the lymph nodes (Jiang et al., 2019). The EEDNV group exhibited a 5.5-fold and 1.3-fold increase in the ratio of mature DCs (CD11c^+^CD80^+^CD86^+^, 26.0 ± 3.5%) compared with the PBS and EDNV groups, respectively (Figure 5g). This finding demonstrated that the nanovesicles significantly enhanced the efficacy of chemo/chemodynamic therapy with EGCG and DOX, thereby promoting DC maturation.

The EEDNV groups exhibited significant tumor infiltration of CD8^+^ T cells compared with the PBS and EENV groups. However, the number of CD8^+^ T cells in the EDNV group was lower than that in the EEDNV group, suggesting that reducing extracellular matrix (ECM) deposition can enhance the tumor infiltration of CD8^+^ T cells (Figure 5h). The EEDNV group demonstrated the highest ratio of tumor-infiltrating effector T cells (CD45^+^CD3^+^CD8^+^IFN-γ^+^), reaching up to 19.9 ± 2.1%, which significantly activated antitumor immunity and prevented tumor inhibition (Supplementary Figure S11).

Tregs play a crucial role in immune suppression by releasing TGF-β1, an immunosuppressive cytokine (Zhang et al., 2019). The typical tumor microenvironment, characterized by low oxygen levels and high lactate concentrations, enhances the immunosuppressive capacity of Tregs (Huang et al., 2022). The ratio of Tregs was 22.8 ± 1.4% in the PBS group and 20.0 ± 5.1% in the EDNV group, which was reduced to 11.4 ± 1.3% and 7.0 ± 0.06% in the EEND and EEDNV groups, respectively, demonstrated that 16 mg/kg EGCG (intravenously administrated once every 3 days for 3 weeks) could slightly inhibit ITM by inhibiting the TGF-β pathway (Supplementary Figure S11). Unsurprisingly, EEDNV resulted in a 4.6-fold increase in the ratio of IFN-γ^+^CD8^+^ T cells to Tregs compared with the EDNV group (Figure 5i), indicating that EEDNV therapy reversed the ITM of TNBC (Yamazaki et al., 2016). In summary, EEDNV has the potential to simultaneously induce mitochondria-mediated ICD effects in tumors, alleviate tumor fibrosis, promote T-cell infiltration, and enhance the antitumor immune response.

## 4 Discussion

Numerous studies have elucidated the precise molecular targets of EGCG in the mETC. Vacca et al. proved that EGCG decreases the levels of subunits of all oxidative phosphorylation (OXPHOS) complexes, such as complexes I, II, IV and V by Western blot (WB) (Valenti et al., 2013). In summary, EGCG improves the hypoxic tumor microenvironment by directly inhibiting the activity of mitochondrial respiratory chain complex I (complex I), blocking electron transfer, reducing ATP production, and increasing ROS levels to trigger the mitochondrial apoptosis pathway (Stevens et al., 2018). Through its chelation with metal ions (e.g., Fe^3+^), EGCG may indirectly affect the function of complex III and modulate oxygen utilization to interfere with complex IV activity, thereby disrupting the electron transport chain, enhancing oxidative stress, and synergizing with chemotherapy drugs to amplify antitumor effects and induce cell death (Liu et al., 2025). Our subsequent research will include experimental validation to further elucidate EGCG’s molecular targeting mechanisms in the mETC.

However, we acknowledge that suboptimal mitochondrial delivery of DOX and/or insufficient immune activation may contribute to residual tumor volume. Specifically, we note that heterogeneous drug distribution within the tumor microenvironment and variable mitochondrial targeting efficiency could limit therapeutic efficacy. Additionally, while EEDNV enhanced immune cell infiltration (Figures 4g, 5h,i), the magnitude may be insufficient for complete tumor regression. To overcome these limitations, there are several key aspects that could be improved by our future work. One approach is to optimize EEDNV’s drug payload or stimuli-responsive release kinetics to boost intratumoral DOX bioavailability (Yao et al., 2024; Zhuang et al., 2024). Moreover, to explore intensified or fractionated dosing regimens to sustain therapeutic drug levels (Xiong et al., 2021; Xiong et al., 2022). Additionally, pairing EEDNV with immune checkpoint inhibitors (e.g., anti-PD-1/PD-L1) to amplify anti-tumor immunity, as preclinical data support synergy between mitochondrial-targeted therapies and immunotherapy (Shang et al., 2023).

EGCG may play a dual role in antitumor immunotherapy, owing to its unique molecular structure. Research has demonstrated that EGCG can activate antitumor immune responses and help overcome immunological tolerance. However, a very high dose of EGCG (2.5 mg/mL aqueous solution taken orally daily for 5 days) has been shown to induce significant immunosuppression (Kang et al., 2007). This immunosuppression may be associated with an inhibitory effect on T cells (Kawai et al., 2004) and/or apoptosis of monocytes (Kawai et al., 2005) triggered by elevated doses of EGCG. As a result, antigen-specific immune responses and antitumor effects elicited by EGCG were only apparent within certain dosage ranges (Kang et al., 2007). One plausible explanation for this phenomenon is that EGCG promotes tumor cell apoptosis, which facilitates the uptake of tumor antigens by specialized APCs and enables cross-presentation in tumor-bearing mice, thereby enhancing the therapeutic effect.

The apoptotic death of tumor cells, which occurs without inflammation, may be perceived as a normal process of tissue turnover, potentially leading to immune ignorance or tolerance of tumor cells (Matzinger, 1994; Sauter et al., 2000; Medzhitov and Janeway, 2002). However, accumulating evidence indicates that in inappropriate immunological environments, apoptotic death of tumor cells induced by cancer therapies can trigger robust antitumor immune responses (Nowak et al., 2003a; Nowak et al., 2003b; Correale et al., 2005). Our previous study demonstrated that a high dose of EGCG (50 μM) induces ROS generation (Zhou et al., 2025). Subsequently, we aimed to determine whether EGCG contributes to ICD in tumor cells at this concentration or under these treatment conditions. In the EENV group of this study, we observed only weak fluorescence intensity of CRT (Figure 5e). Therefore, the complexity of the role of EGCG in antitumor immunotherapy is influenced by its concentration and state during application.

## 5 Conclusion

In this study, we designed and constructed an enzyme-sensitive dePEGylation nanovesicle capable of efficiently loading EGCG and DOX within the phospholipid bilayer (hydrophobic layer) and hydrophilic hollow interior regions through interactions between drugs and nanovesicles. DOX was successfully delivered to the mitochondria by leveraging the mitochondria-targeting properties of EGCG. The rapid removal of the outer PEG shell by MMP-2 facilitated the release of DOX and enhanced the effects of chemotherapy. DOX captured electrons leaking from the mETC by EGCG, leading to the formation of a semiquinone radical of DOX that induced substantial ROS formation and apoptosis. Consequently, EGCG enhanced the therapeutic effect of DOX, particularly by improving the mitochondrial CDT pathway. Additionally, the combination of EGCG and DOX-induced strong mitochondrial-mediated ICD in tumor cells alleviated tumor fibrosis, thereby improving the immunotherapy outcomes of dual-drug-loaded enzyme-sensitive nanovesicles and promoting remission of ITM. This study introduces a novel platform for the development of efficient nanovesicles for cancer immunotherapy and offers new insights into nanovesicle-based cancer treatment strategies.

## Data Availability

The original contributions presented in the study are included in the article/Supplementary Material, further inquiries can be directed to the corresponding authors.

## References

[B1] An H. W. Hou D. Y. Yang J. Wang Z. Q. Wang M. D. Zheng R. (2023). A bispecific glycopeptide spatiotemporally regulates tumor microenvironment for inhibiting bladder cancer recurrence. Sci. Adv. 9, eabq8225. 10.1126/sciadv.abq8225 36857458 PMC9977173

[B2] Chen B. Guo K. Zhao X. Liu Z. Xu C. Zhao N. (2023). Tumor microenvironment-responsive delivery nanosystems reverse immunosuppression for enhanced CO gas/immunotherapy. Exploration 3, 20220140. 10.1002/exp.20220140 38264682 PMC10742199

[B3] Chen C. Ni X. Jia S. Liang Y. Wu X. Kong D. (2019). Massively evoking immunogenic cell death by focused mitochondrial oxidative stress using an AIE luminogen with a twisted molecular structure. Adv. Mater. 31, e1904914. 10.1002/adma.201904914 31696981

[B4] Correale P. Cusi M. G. Del Vecchio M. T. Aquino A. Prete S. P. Tsang K. Y. (2005). Dendritic cell-mediated cross-presentation of antigens derived from Colon carcinoma cells exposed to a highly cytotoxic multidrug regimen with gemcitabine, oxaliplatin, 5-fluorouracil, and leucovorin, elicits a powerful human antigen-specific CTL response with antitumor activity *in vitro* . J. Immunol. 175, 820–828. 10.4049/jimmunol.175.2.820 16002679

[B5] Dou R. Wang L. Zhang J. Cai X. Tang J. Liu X. (2025). Reversing photodynamic therapy-induced tumor metabolic symbiosis and immune evasion delivers a two-punch attack on tumors. Small 21, e2409052. 10.1002/smll.202409052 39950511

[B6] Fontana D. Mauri M. Renso R. Docci M. Crespiatico I. Røst L. M. (2020). ETNK1 mutations induce a mutator phenotype that can be reverted with phosphoethanolamine. Nat. Commun. 11, 5938. 10.1038/s41467-020-19721-w 33230096 PMC7684297

[B7] Fu L. Zhang W. Zhou X. Fu J. He C. (2022). Tumor cell membrane-camouflaged responsive nanoparticles enable MRI-Guided immuno-chemodynamic therapy of orthotopic osteosarcoma. Bioact. Mater. 17, 221–233. 10.1016/j.bioactmat.2022.01.035 35386464 PMC8965157

[B8] Gao P. Sun X. Chen X. Wang Y. Foster B. A. Subjeck J. (2008). Secretable chaperone Grp170 enhances therapeutic activity of a novel tumor suppressor, mda-7/IL-24. Cancer Res. 68, 3890–3898. 10.1158/0008-5472.Can-08-0156 18483274 PMC3025602

[B9] Guo J. Yu Z. Sun D. Zou Y. Liu Y. Huang L. (2021). Two nanoformulations induce reactive oxygen species and immunogenetic cell death for synergistic chemo-immunotherapy eradicating colorectal cancer and hepatocellular carcinoma. Mol. Cancer 20, 10. 10.1186/s12943-020-01297-0 33407548 PMC7786897

[B10] Guo Y. Zhang Q. Zhu Q. Gao J. Zhu X. Yu H. (2022). Copackaging photosensitizer and PD-L1 siRNA in a nucleic acid nanogel for synergistic cancer photoimmunotherapy. Sci. Adv. 8, eabn2941. 10.1126/sciadv.abn2941 35442728 PMC9020667

[B11] Hanoteau A. Newton J. M. Krupar R. Huang C. Liu H. C. Gaspero A. (2019). Tumor microenvironment modulation enhances immunologic benefit of chemoradiotherapy. J. Immunother. Cancer 7, 10. 10.1186/s40425-018-0485-9 30646957 PMC6332704

[B12] He Y. Lei L. Cao J. Yang X. Cai S. Tong F. (2021). A combinational chemo-immune therapy using an enzyme-sensitive nanoplatform for dual-drug delivery to specific sites by Cascade targeting. Sci. Adv. 7, eaba0776. 10.1126/sciadv.aba0776 33547067 PMC7864565

[B13] Hou B. Ye J. Huang L. Cheng W. Chen F. Zhou H. (2024). Tumor-specific delivery of clickable inhibitor for PD-L1 degradation and mitigating resistance of radioimmunotherapy. Sci. Adv. 10, eadq3940. 10.1126/sciadv.adq3940 39546592 PMC11567003

[B14] Huang M. Xiong D. Pan J. Zhang Q. Wang Y. Myers C. R. (2022). Prevention of tumor growth and dissemination by *in situ* vaccination with mitochondria-targeted atovaquone. Adv. Sci. 9, e2101267. 10.1002/advs.202101267 PMC9036031 35243806

[B15] Jana D. Zhao Y. (2022). Strategies for enhancing cancer chemodynamic therapy performance. Exploration 2, 20210238. 10.1002/EXP.20210238 37323881 PMC10191001

[B16] Jia H. Liu J. Fang T. Zhou Z. Li R. Yin W. (2023). The role of altered lipid composition and distribution in liver fibrosis revealed by multimodal nonlinear optical microscopy. Sci. Adv. 9, eabq2937. 10.1126/sciadv.abq2937 36638165 PMC9839333

[B17] Jiang W. Yin L. Chen H. Paschall A. V. Zhang L. Fu W. (2019). NaCl nanoparticles as a cancer therapeutic. Adv. Mater. 31, e1904058. 10.1002/adma.201904058 31553099 PMC6886716

[B18] Kang T. H. Lee J. H. Song C. K. Han H. D. Shin B. C. Pai S. I. (2007). Epigallocatechin-3-gallate enhances CD8+ T cell-mediated antitumor immunity induced by DNA vaccination. Cancer Res. 67, 802–811. 10.1158/0008-5472.Can-06-2638 17234792 PMC3181129

[B19] Kawai K. Tsuno N. H. Kitayama J. Okaji Y. Yazawa K. Asakage M. (2004). Epigallocatechin gallate attenuates adhesion and migration of CD8+ T cells by binding to CD11b. J. Allergy Clin. Immunol. 113, 1211–1217. 10.1016/j.jaci.2004.02.044 15208607

[B20] Kawai K. Tsuno N. H. Kitayama J. Okaji Y. Yazawa K. Asakage M. (2005). Epigallocatechin gallate induces apoptosis of monocytes. J. Allergy Clin. Immunol. 115, 186–191. 10.1016/j.jaci.2004.10.005 15637567

[B21] Li Q. Chen C. Kong J. Li L. Li J. Huang Y. (2022). Stimuli-responsive nano vehicle enhances cancer immunotherapy by coordinating mitochondria-targeted immunogenic cell death and PD-L1 blockade. Acta Pharm. Sin. B 12, 2533–2549. 10.1016/j.apsb.2021.11.005 35646521 PMC9136536

[B22] Liu H. Hu Y. Sun Y. Wan C. Zhang Z. Dai X. (2019). Co-delivery of bee venom melittin and a photosensitizer with an organic-inorganic hybrid nanocarrier for photodynamic therapy and immunotherapy. ACS Nano 13, 12638–12652. 10.1021/acsnano.9b04181 31625721

[B23] Liu J. Zhou Y. Lyu Q. Yao X. Wang W. (2024). Targeted protein delivery based on stimuli-triggered nanomedicine. Exploration 4, 20230025. 10.1002/exp.20230025 38939867 PMC11189579

[B24] Liu T. Xiong C. F. Zhang L. J. Jiao G. H. Shi H. Feng J. (2023). Boosting doxorubicin-induced mitochondria apoptosis for the monodrug-mediated combination of chemotherapy and chemodynamic therapy. Adv. Healthc. Mater. 12, e2202045. 10.1002/adhm.202202045 36239177

[B25] Liu Z. Yang Y. Kong X. Ren X. Xuan F. (2025). Drug-device-field integration for mitochondria-targeting dysfunction and tumor therapy by home-tailored pyroelectric nanocomposites. Biomaterials 316, 122990. 10.1016/j.biomaterials.2024.122990 39637584

[B26] Lu Y. Zeng T. Zhang H. Li Y. Zhu X. Liu H. (2023). Nano-immunotherapy for lung cancer. Nano TransMed 2, e9130018. 10.26599/NTM.2023.9130018

[B27] Luo J. Wang X. Shi Z. Zeng Y. He L. Cao J. (2022). Enhancement of antitumor immunotherapy using mitochondria-targeted cancer cell membrane-biomimetic MOF-Mediated sonodynamic therapy and checkpoint blockade immunotherapy. J. Nanobiotechnology 20, 228. 10.1186/s12951-022-01453-2 35568916 PMC9107704

[B28] Marigo I. Zilio S. Desantis G. Mlecnik B. Agnellini A. H. R. Ugel S. (2016). T cell cancer therapy requires CD40-CD40L activation of tumor necrosis factor and inducible nitric-oxide-synthase-producing dendritic cells. Cancer Cell 30, 651–390. 10.1016/j.ccell.2016.09.009 27728809

[B29] Matzinger P. (1994). Tolerance, danger, and the extended family. Annu. Rev. Immunol. 12, 991–1045. 10.1146/annurev.iy.12.040194.005015 8011301

[B30] Medzhitov R. Janeway C. A. Jr. (2002). Decoding the patterns of self and nonself by the innate immune system. Science 296, 298–300. 10.1126/science.1068883 11951031

[B31] Nagakubo T. Kumano T. Ohta T. Hashimoto Y. Kobayashi M. (2019). Copper amine oxidases catalyze the oxidative deamination and hydrolysis of cyclic imines. Nat. Commun. 10, 413. 10.1038/s41467-018-08280-w 30679427 PMC6345859

[B32] Nam G. H. Lee E. J. Kim Y. K. Hong Y. Choi Y. Ryu M. J. (2018). Combined rho-kinase inhibition and immunogenic cell death triggers and propagates immunity against cancer. Nat. Commun. 9, 2165. 10.1038/s41467-018-04607-9 29867097 PMC5986820

[B33] Ni K. Luo T. Nash G. T. Lin W. (2020). Nanoscale metal-organic frameworks for cancer immunotherapy. Acc. Chem. Res. 53, 1739–1748. 10.1021/acs.accounts.0c00313 32808760 PMC9359629

[B34] Nowak A. K. Lake R. A. Marzo A. L. Scott B. Heath W. R. Collins E. J. (2003a). Induction of tumor cell apoptosis *in vivo* increases tumor antigen cross-presentation, cross-priming rather than cross-tolerizing host tumor-specific CD8 T cells. J. Immunol. 170, 4905–4913. 10.4049/jimmunol.170.10.4905 12734333

[B35] Nowak A. K. Robinson B. W. Lake R. A. (2003b). Synergy between chemotherapy and immunotherapy in the treatment of established murine solid tumors. Cancer Res. 63, 4490–4496. 10.1097/00002820-200308000-00012 12907622

[B36] Pan J. Lai Y. Zhang S. Zhang H. Shan Y. Huang L. (2023). Self-adaptive nanoregulator to mitigate dynamic immune evasion of pancreatic cancer. Adv. Mater. 35, e2305798. 10.1002/adma.202305798 37716012

[B37] Pan K. Farrukh H. Chittepu V. Xu H. Pan C. X. Zhu Z. (2022). CAR race to cancer immunotherapy: from CAR T, CAR NK to CAR macrophage therapy. J. Exp. Clin. Cancer Res. 41, 119. 10.1186/s13046-022-02327-z 35361234 PMC8969382

[B38] Peng H. Yao F. Zhao J. Zhang W. Chen L. Wang X. (2023). Unraveling mitochondria-targeting reactive oxygen species modulation and their implementations in cancer therapy by nanomaterials. Exploration 3, 20220115. 10.1002/exp.20220115 37324035 PMC10191003

[B39] Pinter M. Jain R. K. (2017). Targeting the renin-angiotensin system to improve cancer treatment: implications for immunotherapy. Sci. Transl. Med. 9, eaan5616. 10.1126/scitranslmed.aan5616 28978752 PMC5928511

[B40] Qian K. Gao S. Jiang Z. Ding Q. Cheng Z. (2024). Recent advances in mitochondria-targeting Theranostic agents. Exploration 4, 20230063. 10.1002/exp.20230063 39175881 PMC11335472

[B41] Riley R. S. June C. H. Langer R. Mitchell M. J. (2019). Delivery technologies for cancer immunotherapy. Nat. Rev. Drug Discov. 18, 175–196. 10.1038/s41573-018-0006-z 30622344 PMC6410566

[B42] Sauter B. Albert M. L. Francisco L. Larsson M. Somersan S. Bhardwaj N. (2000). Consequences of cell death: exposure to necrotic tumor cells, but not primary tissue cells or apoptotic cells, induces the maturation of immunostimulatory dendritic cells. J. Exp. Med. 191, 423–434. 10.1084/jem.191.3.423 10662788 PMC2195816

[B43] Shang Y. Lu H. Liao L. Li S. Xiong H. Yao J. (2023). Bioengineered nanospores selectively blocking LC3-Associated phagocytosis in tumor-associated macrophages potentiate antitumor immunity. ACS Nano 17, 10872–10887. 10.1021/acsnano.3c02657 37192052

[B44] Shi Y. Wu Y. Li F. Jiang K. Fang X. Wang Y. (2024). Investigating the immunogenic cell death-dependent subtypes and prognostic signature of triple-negative breast cancer. Phenomics 4, 34–45. 10.1007/s43657-023-00133-x 38605910 PMC11003942

[B45] Siedlar A. M. Seredenina T. Faivre A. Cambet Y. Stasia M. J. André-Lévigne D. (2023). NADPH oxidase 4 is dispensable for skin myofibroblast differentiation and wound healing. Redox Biol. 60, 102609. 10.1016/j.redox.2023.102609 36708644 PMC9950659

[B46] Smith M. R. Chacko B. K. Johnson M. S. Benavides G. A. Uppal K. Go Y. M. (2020). A precision medicine approach to defining the impact of doxorubicin on the bioenergetic-metabolite interactome in human platelets. Redox Biol. 28, 101311. 10.1016/j.redox.2019.101311 31546171 PMC6812033

[B47] Stevens J. F. Revel J. S. Maier C. S. (2018). Mitochondria-centric review of polyphenol bioactivity in cancer models. Antioxid. Redox Signal. 29, 1589–1611. 10.1089/ars.2017.7404 29084444 PMC6207154

[B48] Trujillo J. A. Luke J. J. Zha Y. Segal J. P. Ritterhouse L. L. Spranger S. (2019). Secondary resistance to immunotherapy associated with β-catenin pathway activation or PTEN loss in metastatic melanoma. J. Immunother. Cancer 7, 295. 10.1186/s40425-019-0780-0 31703593 PMC6839232

[B49] Valenti D. De Bari L. Manente G. A. Rossi L. Mutti L. Moro L. (2013). Negative modulation of mitochondrial oxidative phosphorylation by epigallocatechin-3 gallate leads to growth arrest and apoptosis in human malignant pleural mesothelioma cells. Biochim. Biophys. Acta 1832, 2085–2096. 10.1016/j.bbadis.2013.07.014 23911347

[B50] Wang F. Xu D. Su H. Zhang W. Sun X. Monroe M. K. (2020). Supramolecular prodrug hydrogelator as an immune booster for checkpoint blocker-based immunotherapy. Sci. Adv. 6, eaaz8985. 10.1126/sciadv.aaz8985 32490201 PMC7239700

[B51] Wei Y. Kim T. J. Peng D. H. Duan D. Gibbons D. L. Yamauchi M. (2017). Fibroblast-specific inhibition of TGF-β1 signaling attenuates lung and tumor fibrosis. J. Clin. Invest. 127, 3675–3688. 10.1172/JCI94624 28872461 PMC5617667

[B52] Wu H. Yu M. Miao Y. He S. Dai Z. Song W. (2019). Cholesterol-tuned liposomal membrane rigidity directs tumor penetration and anti-tumor effect. Acta Pharm. Sin. B 9, 858–870. 10.1016/j.apsb.2019.02.010 31384544 PMC6664103

[B53] Xiong H. Liu X. Xie Z. Zhu L. Lu H. Wang C. (2022). Metabolic symbiosis-blocking nano-combination for tumor vascular normalization treatment. Adv. Healthc. Mater. 11, 2102724. 10.1002/adhm.202102724 35708141

[B54] Xiong H. Wang C. Wang Z. Lu H. Yao J. (2021). Self-assembled nano-activator constructed ferroptosis-immunotherapy through hijacking endogenous iron to intracellular positive feedback loop. J. Control. Release 332, 539–552. 10.1016/j.jconrel.2021.03.007 33689796

[B55] Yamazaki T. Pitt J. M. Vétizou M. Marabelle A. Flores C. Rekdal Ø. (2016). The oncolytic peptide LTX-315 overcomes resistance of cancers to immunotherapy with CTLA4 checkpoint blockade. Cell Death Differ. 23, 1004–1015. 10.1038/cdd.2016.35 27082453 PMC4987735

[B56] Yang T. Hu Y. Miao J. Chen J. Liu J. Cheng Y. (2022). A BRD4 PROTAC nanodrug for glioma therapy *via* the intervention of tumor cells proliferation, apoptosis and M2 macrophages polarization. Acta Pharm. Sin. B 12, 2658–2671. 10.1016/j.apsb.2022.02.009 35755286 PMC9214068

[B57] Yao L. Zhu X. Shan Y. Zhang L. Yao J. Xiong H. (2024). Recent progress in anti‐tumor nanodrugs based on tumor microenvironment redox regulation. Small 20, e2310018. 10.1002/smll.202310018 38269480

[B58] Zhang Y. Bush X. Yan B. Chen J. A. (2019). Gemcitabine nanoparticles promote antitumor immunity against melanoma. Biomaterials 189, 48–59. 10.1016/j.biomaterials.2018.10.022 30388589 PMC6281175

[B59] Zhao C. Zheng T. Wang R. Lin X. Hu Z. Zhao Z. (2024). Synergistically augmenting cancer immunotherapy by physical manipulation of pyroptosis induction. Phenomics 4, 298–312. 10.1007/s43657-023-00140-y 39398428 PMC11466912

[B60] Zheng Y. Han Y. Sun Q. Li Z. (2022). Harnessing anti-tumor and tumor-tropism functions of macrophages *via* nanotechnology for tumor immunotherapy. Exploration 2, 20210166. 10.1002/exp.20210166 37323705 PMC10190945

[B61] Zhou M. Wang J. Pan J. Wang H. Huang L. Hou B. (2023b). Nanovesicles loaded with a TGF-β receptor 1 inhibitor overcome immune resistance to potentiate cancer immunotherapy. Nat. Commun. 14, 3593. 10.1038/s41467-023-39035-x 37328484 PMC10275881

[B62] Zhou M. Zhou C. Geng H. Huang Z. Lin Z. Wang Y. (2025). EGCG-Enabled deep tumor penetration of phosphatase and acidity dual-responsive nanotherapeutics for combinatory therapy of breast cancer. Small 21, 2406245. 10.1002/smll.202406245 39558766

[B63] Zhou M.-X. Zhang J.-Y. Cai X.-M. Dou R. Ruan L.-F. Yang W.-J. (2023a). Tumor-penetrating and mitochondria-targeted drug delivery overcomes doxorubicin resistance in lung cancer. Chin. J. Polym. Sci. 41, 525–537. 10.1007/s10118-022-2775-4

[B64] Zhu Y. Zhang S. Lai Y. Pan J. Chen F. Wang T. (2022). Self-cooperative prodrug nanovesicles migrate immune evasion to potentiate chemoradiotherapy in head and neck cancer. Adv. Sci. 9, e2203263. 10.1002/advs.202203263 PMC9798966 36344430

[B65] Zhuang H. Wang R. Qi Y. Liu Y. Xiong H. Yao J. (2024). Nanocoated bacteria with H2S generation-triggered self-amplified photothermal and photodynamic effect for breast cancer therapy. J. Control. Release 373, 507–519. 10.1016/j.jconrel.2024.07.036 39025267

