# Supplementary material for: EGCG and DOX dual-drug-loaded enzyme-responsive nanovesicles boost mitochondrial-mediated ICD for improved immunotherapy
Source: Front Pharmacol. 2025 Jul 7;16:1624109. doi: 10.3389/fphar.2025.1624109 (PMC12277346; doi:10.3389/fphar.2025.1624109)
Supplement: Supplementary file 1 [file Supplementaryfile1.docx]

Supplementary Material

# Supplementary Figures and Tables


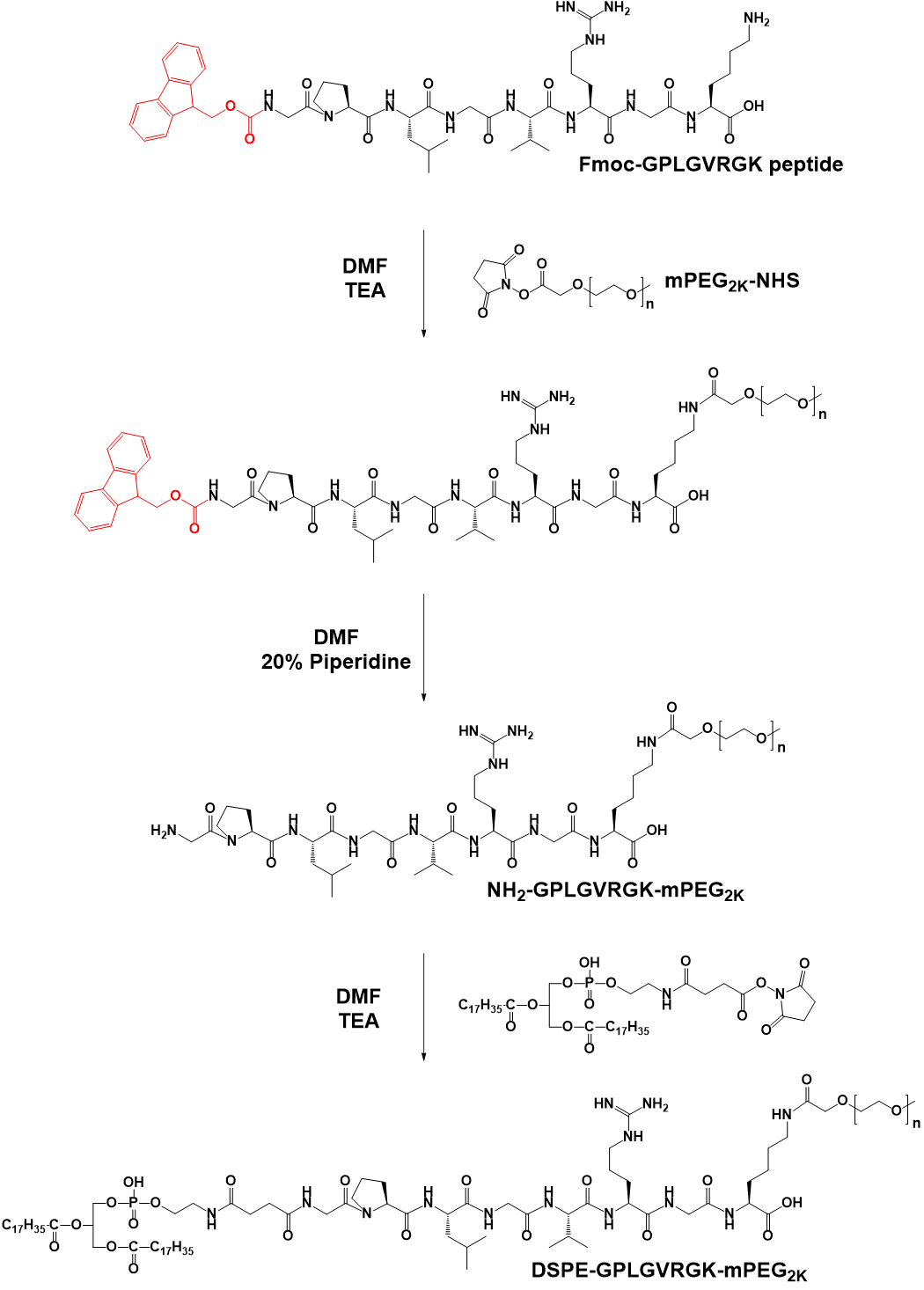


**Supplementary Figure 1.** Synthesis of DSPE-GPLGVRGK-mPEG_2K_.


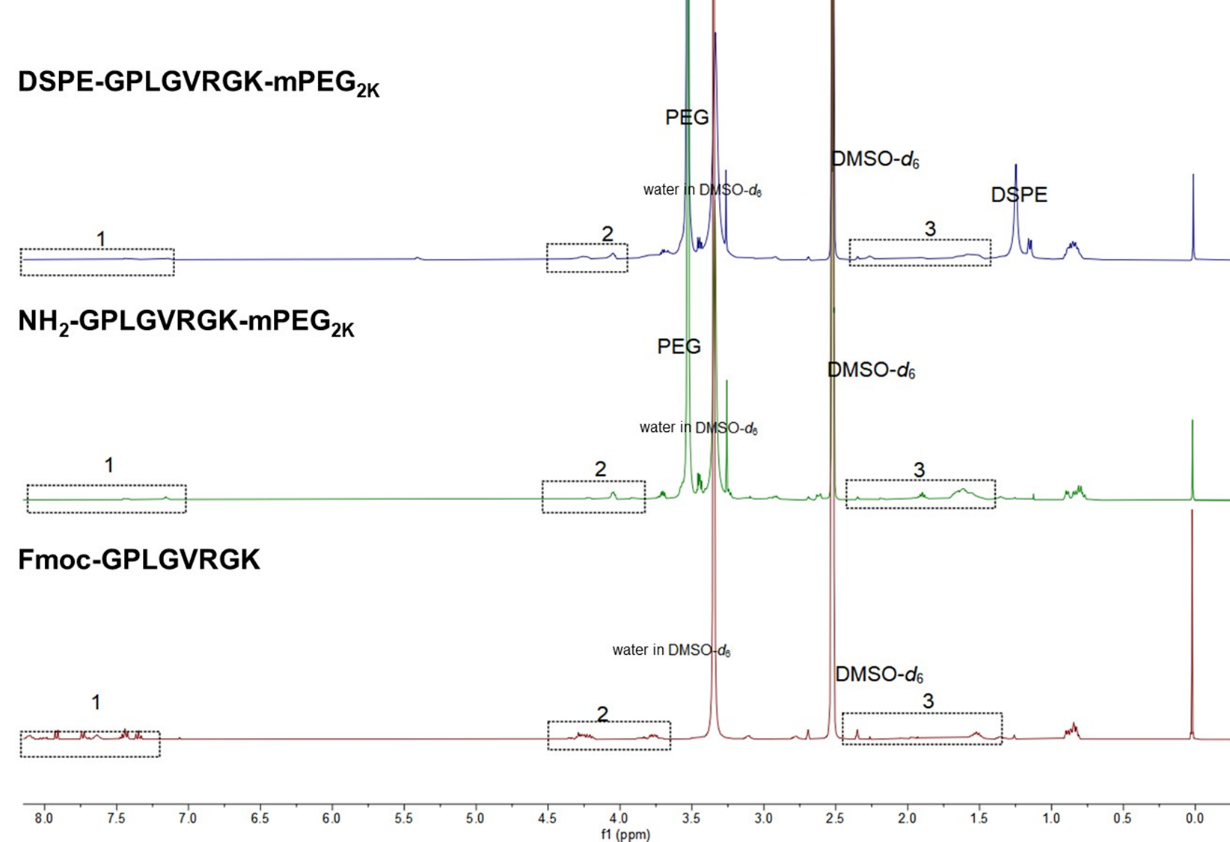


**Supplementary Figure 2.** ^1^H NMR spectra of DSPE-GPLGVRGK-mPEG_2K_ in DMSO-d_6_.


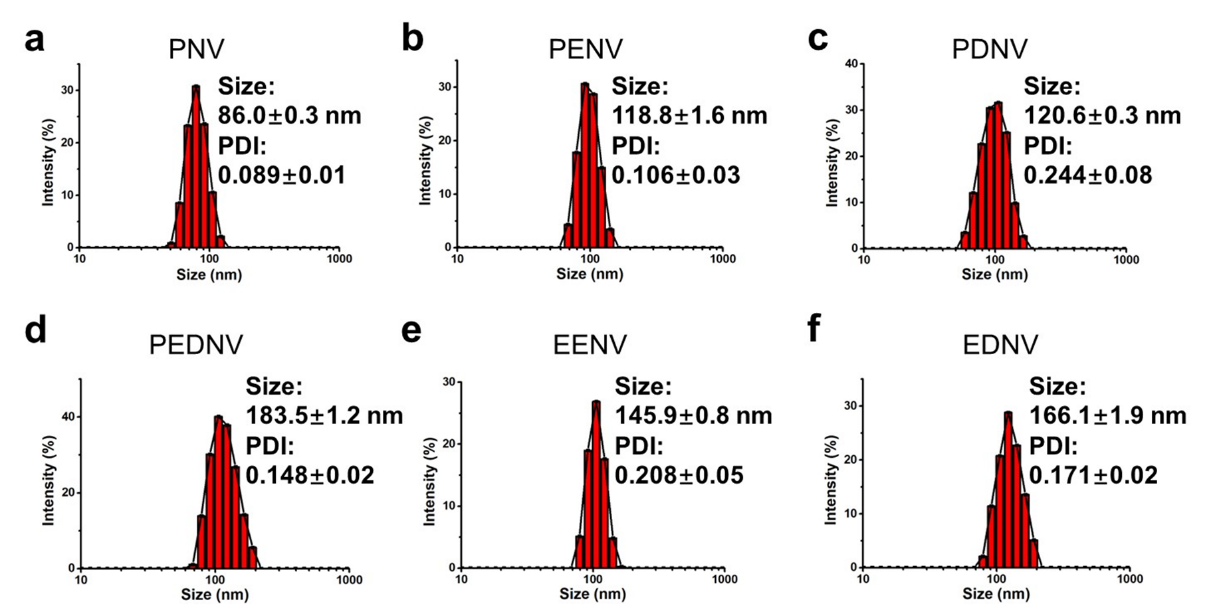


**Supplementary Figure 3.** Particle size distribution of nanovesicles determined by DLS. All data are presented as mean ± SD. *n* = 3 independent experiments.

**Supplementary Table 1.** Zeta potentials of different nanovesicles. All data are presented as mean ± SD. *n* = 3 independent experiments.

| **MMP-2-insensitive nanovesicles** | | **MMP-2-sensitive nanovesicles** | |
| --- | --- | --- | --- |
| **groups** | **ζ-potential (mV)** | **groups** | **ζ-potential (mV)** |
| ENV | -8.46±0.68 | PNV | -5.73±0.82 |
| EENV | -12.51±0.59 | PENV | -10.39±1.54 |
| EDNV | +7.96±0.87 | PDNV | +8.63±1.08 |
| EEDNV | -2.15±0.05 | PEDNV | -3.80±0.63 |

**Supplementary Table 2.** Drug loading and encapsulation rates of DOX and EGCG. All data are presented as mean ± SD. *n* = 3 independent experiments. DL drug loading, EE encapsulation efficiency.

| **EGCG** | | | | **DOX** | | |
| --- | --- | --- | --- | --- | --- | --- |
| **groups** | **EE (%)** | **DL (%)** | **groups** | | **EE (%)** | **DL (%)** |
| EENV | 95.7±0.07 | 16.3±0.1 | EDNV | | 80.2±3.54 | 1.60±0.04 |
| PENV | 98.2±0.26 | 16.6±0.4 | PDNV | | 79.6±0.15 | 1.59±0.17 |
| EEDNV | 96.1±0.40 | 16.1±0.3 | EEDNV | | 69.1±1.31 | 1.16±0.04 |
| PEDNV | 97.5±0.15 | 16.3±0.7 | PEDNV | | 70.9±2.65 | 1.19±0.06 |


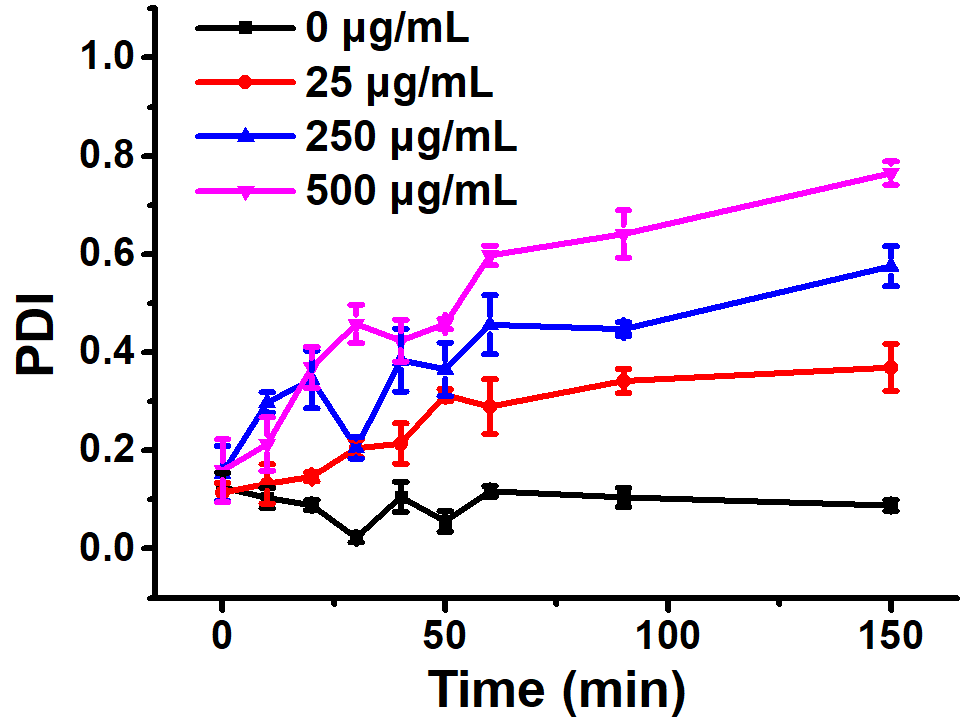


**Supplementary Figure 4.** Changes in PDI of EEDNV after incubation with MMP-2 at different times. All data are presented as mean ± SD. *n* = 3 independent experiments.


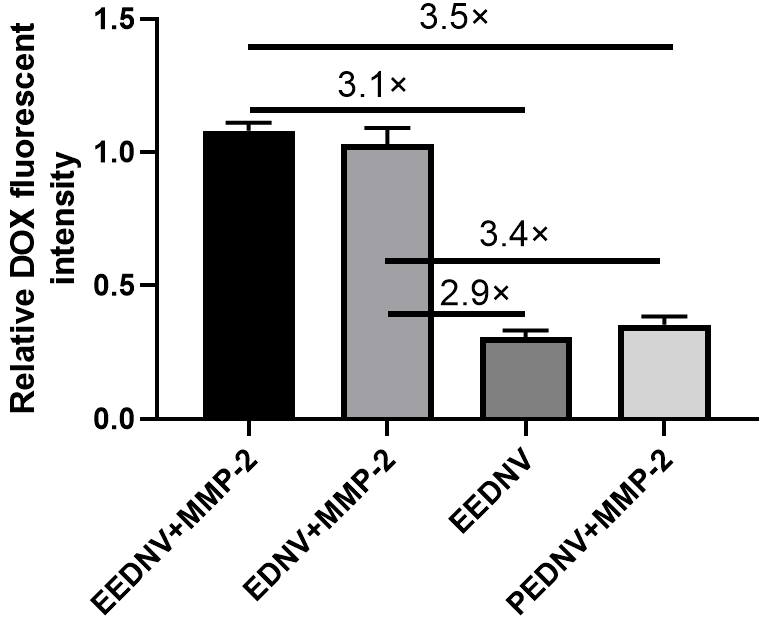


**Supplementary Figure 5**. Quantitation of fluorescence intensity as a measure of nanovesicles uptake in 4T1 cells.


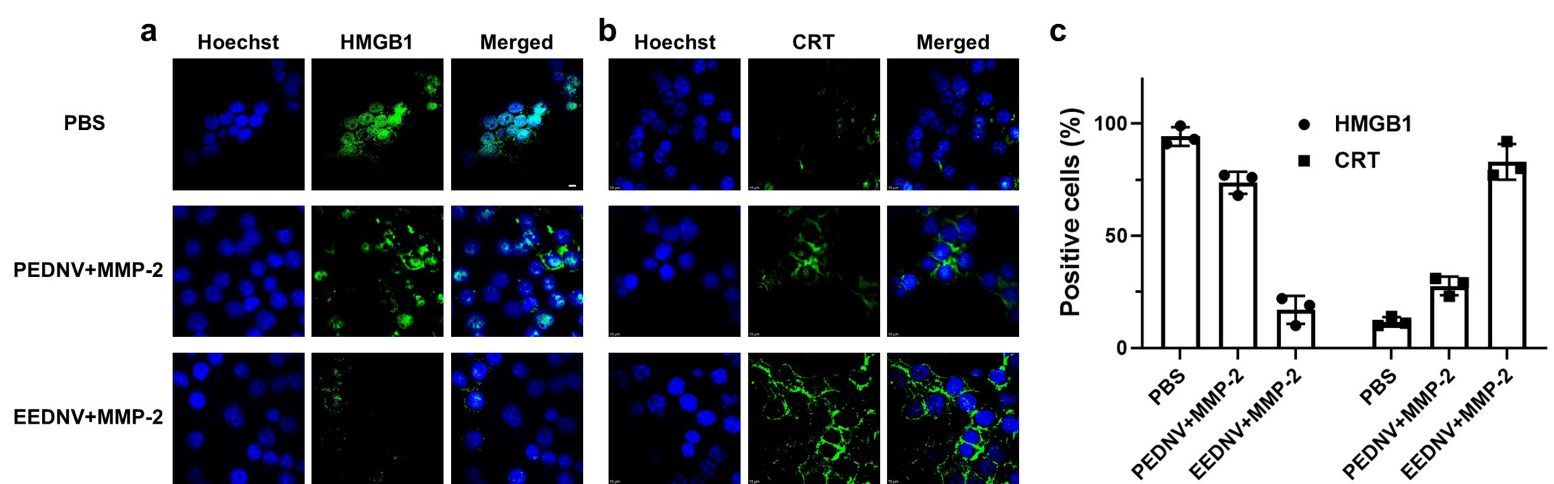


**Supplementary Figure 6**. (a, b) CLSM images of EEDNV-mediated extracellular efflux of HMGB1 release and CRT exposure on the membrane of the tumor cells (Scale bar = 10 μm). The experiment was repeated independently 3 times with similar results. (c) Quantify the CRT exposure and HMGB1 efflux in 4T1 cells after treatment with EEDNV (n = 3 biologically independent samples).


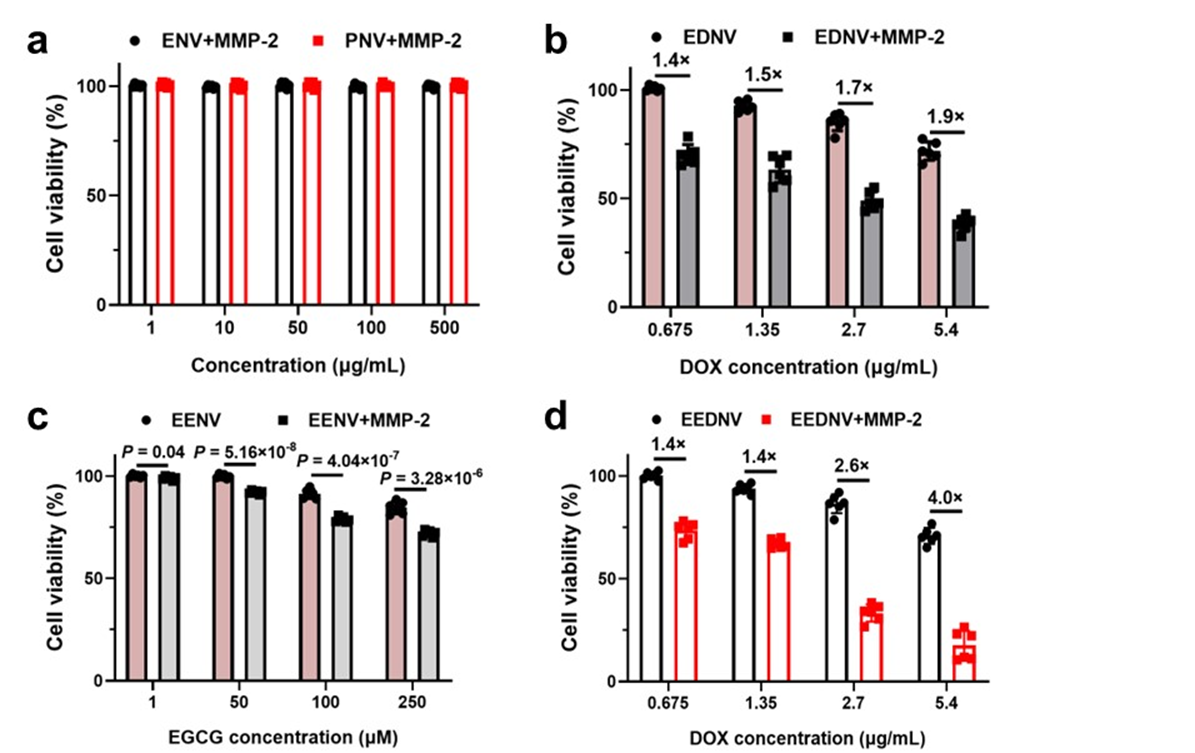


**Supplementary Figure 7.** *In vitro* cytotoxicity of different nanovesicles towards 4T1 cells. Cell viabilities of (**a**) blank nanovesicles with MMP-2, (**b**) DOX-loaded enzyme-sensitive nanovesicles with or without MMP-2, (**c**) EGCG-loaded enzyme-sensitive nanovesicles with or without MMP-2 and (**d**) dual drug-loaded enzyme-sensitive nanovesicles with or without MMP-2 (mean ± SD, *n* = 6 biologically independent cells).


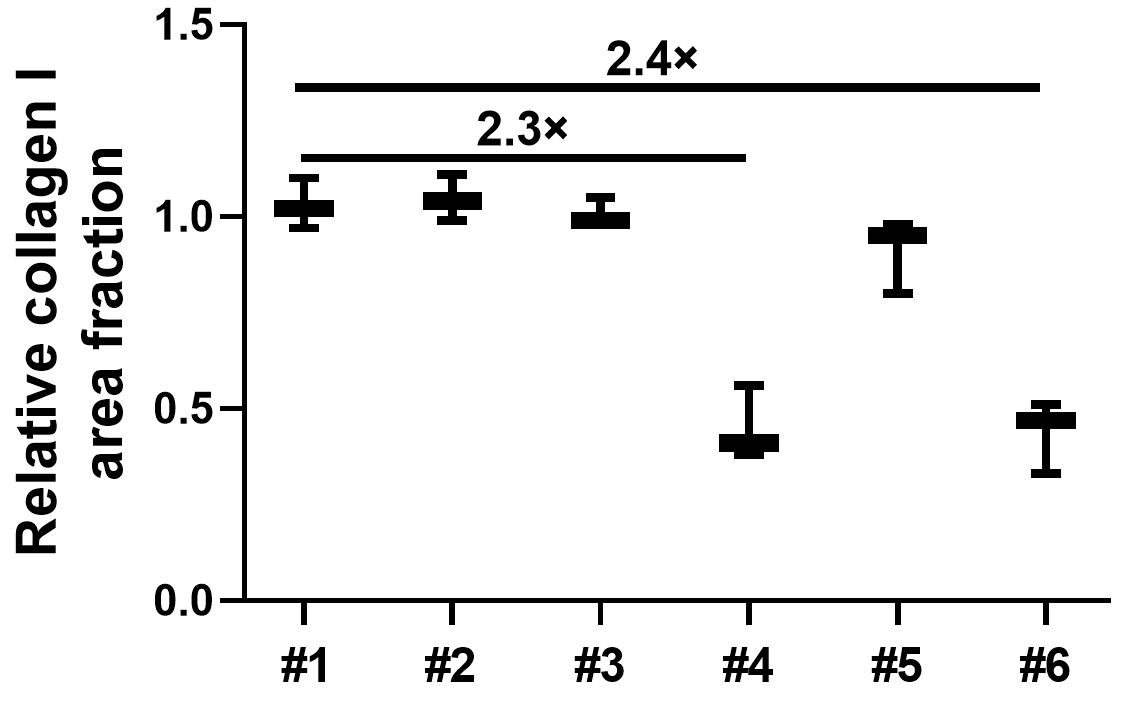


**Supplementary Figure 8.** Semi-quantitative analysis of relative collagen I area fraction in Figure 4g (n = 3 mice). Error bars represent mean ± SD.


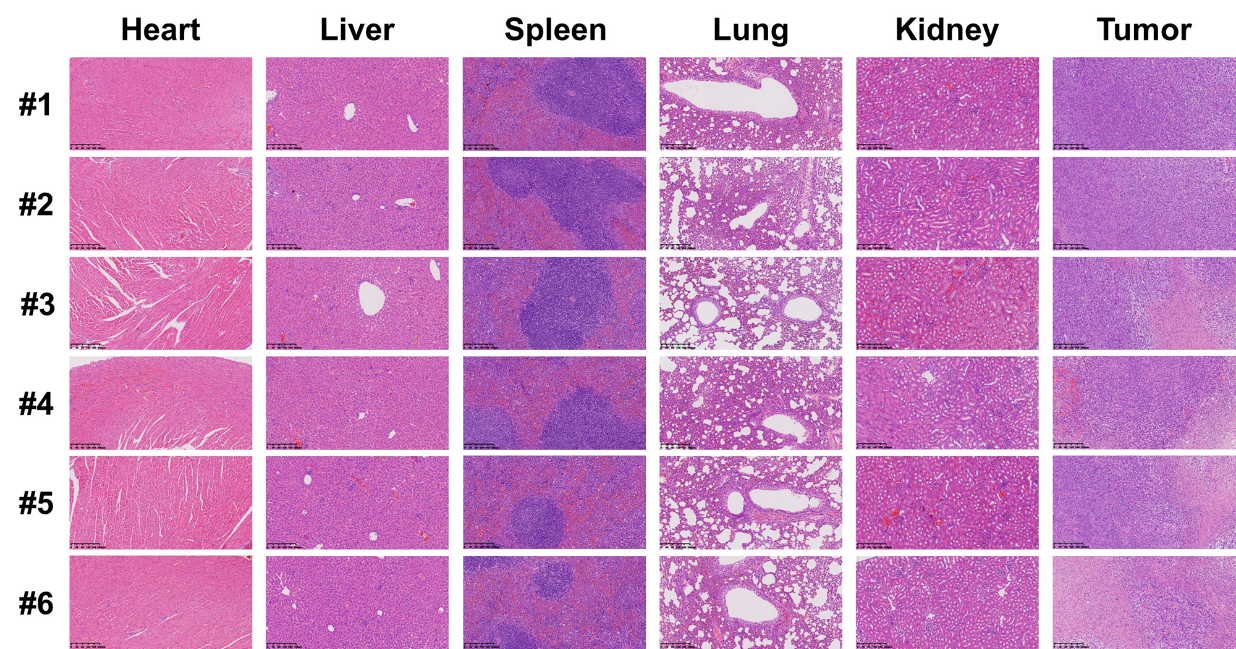


**Supplementary Figure 9.** H&E staining of typical organs at the end of different treatments. Scale bar=200 μm.

**
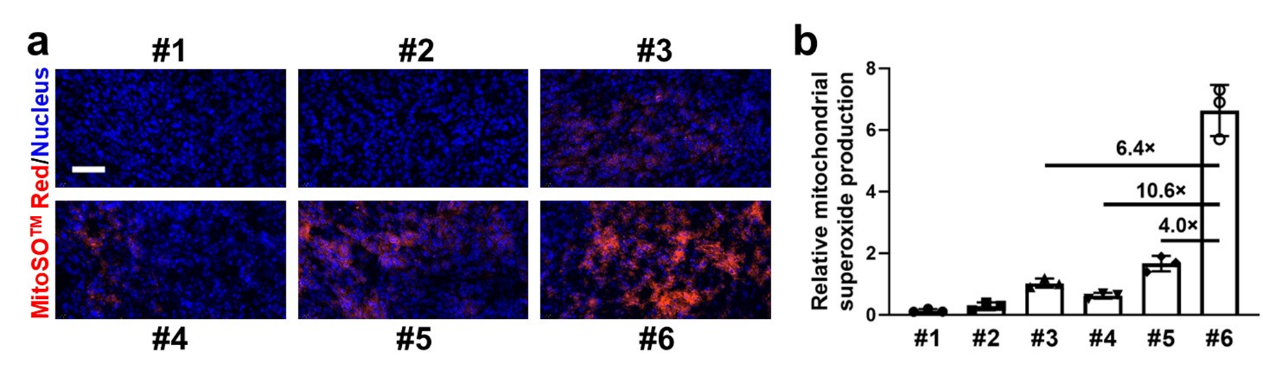
**

**Supplementary Figure 10.** Representative images of MitoSO^TM^ Red staining and intensity of MitoSO^TM^ Red staining (n=3 mice). (**a**) Fluorescence images of the tissue sections stained with MitoSO^TM^ Red upon different treatments. Scale bar = 50 μm. (**b**) Semi-quantitative analysis of the relative mitochondrial superoxide production in (a) by Image J. Error bars represent mean ± SD. The experiment was repeated independently 3 times with similar results.


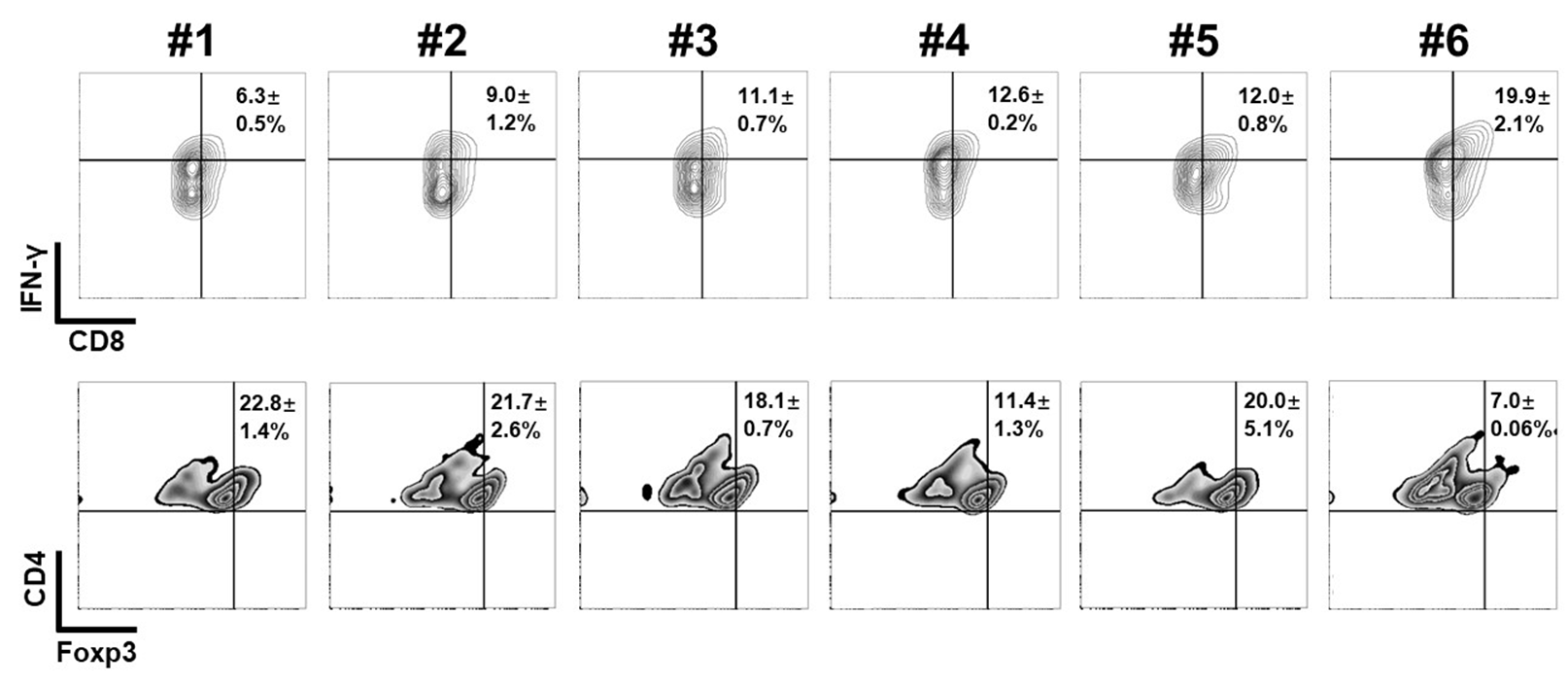


**Supplementary Figure 11.** Immunological evaluation of different nanovesicle-based immunotherapies for TNBC. Flow cytometric quantification of intratumoral infiltration of IFN-γ^+^CD8^+^ T cells (CD3^+^CD8^+^IFN-γ^+^) and Tregs (CD3^+^CD4^+^Foxp3^+^) in NIH3T3/4T1 tumor model. All data are presented as mean ± SD. *n* = 3 mice.
